# The m6A Demethylase Fto Enhances Susceptibility to Atrial Fibrillation by Demethylating *Kcne1* in Aging Mice

**DOI:** 10.1111/acel.70263

**Published:** 2025-10-12

**Authors:** Ruopeng Tan, Yuanjun Sun, Mengyang Yuan, Lin Wang, Xinyu Yang, Genlong Xue, Guiwen Xu, Yang Liu, Xiaomeng Yin

**Affiliations:** ^1^ Institute of Cardiovascular Diseases The First Affiliated Hospital of Dalian Medical University Dalian China; ^2^ Department of Cardiology The First Affiliated Hospital of Dalian Medical University Dalian China

**Keywords:** aging, atrial fibrillation, demethylase, m6A methylation modification

## Abstract

Aging is a risk factor for atrial fibrillation (AF). In 19‐month‐old mice, increases in AF inducibility are associated with enhanced protein levels of fat mass and obesity‐associated protein (Fto), and reduced N6‐methyladenosine (m6A) modification in atrial tissue. Whether Fto‐regulated m6A demethylation is involved in aging‐induced AF remains unclear. AF inducibility and electrophysiology were performed through programmed stimulation and optical mapping. The intensities of slow delayed rectifier potassium currents (IKs) were measured by patch‐clamp. m6A‐sequencing revealed that Kcne1 mRNA was m6A‐demethylated in aging mouse atria. Kcne1 knockdown in 2‐month‐old mice increased AF inducibility. Aging mice with cardiomyocyte‐specific Fto knockout had increased Kcne1 mRNA and protein levels, with reduced susceptibility to AF. Additionally, overexpression of wild‐type Fto, rather than a catalytically inactive mutant in 2‐month‐old mice, reduced Kcne1 protein levels, leading to enhanced IKs current and AF inducibility. Furthermore, the negative relationship between FTO and KCNE1 was confirmed in left atrial appendage samples from AF patients. In iPSC‐derived atrial cardiomyocytes, FTO‐mediated KCNE1 demethylation repressed KCNE1 pre‐mRNA splicing, mRNA nuclear export, and translational efficacy. Collectively, aging‐induced elevation of Fto represses m6A methylation of Kcne1, which in turn leads to reductions in Kcne1 mRNA and protein levels in atrial cardiomyocytes, thereby increasing AF inducibility.

## Graphical abstract

Aging‐induced accumulation of PGE2 in cardiomyocytes blocks the degradation of Fto. Elevation of Fto in the atria represses m6A methylation of Kcnel which, in turn, leads to the reduction of Kcnel mRNA and protein. Reduced Kcnel enhances IKs currents and thereby shortens APD in atrial cardiomyocytes and increases AF inducibility.

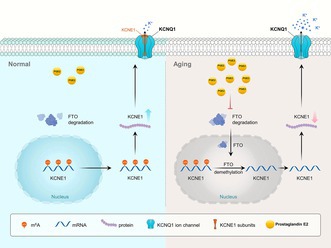

## 1 Introduction

Atrial fibrillation (AF) is a common arrhythmia characterized by an irregular and rapid heartbeat. Its prevalence increases with age (Wilke et al. 2013; Schnabel et al. 2015; Feinberg et al. 1995), which is an independent risk factor for adverse outcomes (Hijazi et al. 2016; Marinigh et al. 2010; Feinberg et al. 1995). Although catheter ablation is effective, elderly patients face higher recurrence rates (Bunch et al. 2016). The mechanisms underlying aging‐related AF remain poorly understood, hampering therapeutic development.

RNA modifications play essential roles in regulating post‐transcriptional gene expression, influencing mRNA stability, localization, and translation. Among more than 140 known chemical modifications on RNA, N6‐methyladenosine (m6A) is one of the most abundant and dynamically regulated (Zhang et al. 2018). It has been demonstrated to be critical for cardiac development, function, and stress responses (Gao et al. 2020; Mathiyalagan et al. 2019; Dorn et al. 2019). Notably, recent studies indicate that m6A modification is involved in the aging process and age‐related diseases (Wu et al. 2023). Decreased m6A modification has been shown to promote senescence‐associated beta‐galactosidase activity and lipid accumulation in the skeletal muscle of aging cynomolgus monkeys (Wu et al. 2023). Conversely, pharmacological inhibition of systemic m6A levels markedly upregulates the expression of neuroinflammatory genes in the brains of SAMP8 knockout‐induced aging mice (Irisarri et al. 2025). The deposition and removal of m6A are catalyzed by methyltransferases (such as METTL3, METTL14, and WTAP) and demethylases (including FTO and ALKBH5), respectively (Yang et al. 2018). FTO has been demonstrated to influence metabolic traits and cardiac pathophysiology (Mathiyalagan et al. 2019), with growing evidence indicating its involvement in the regulation of aging (Krejčí et al. 2023).

Cardiac action potentials are tightly regulated by ion channel complexes. KCNQ1, when assembled with its regulatory subunit KCNE1, mediates the slowly activating delayed rectifier potassium current (IKs) (Barhanin et al. 1996; Sanguinetti et al. 1996; Marx et al. 2002), which is critical for myocardial repolarization. KCNE1 is highly expressed in the heart (Cui 2016; Robbins 2001) and modulates KCNQ1 channel gating and kinetics. Loss of KCNE1 function shortens the action potential duration and increases susceptibility to atrial arrhythmias, including AF (Temple et al. 2005). Recent advances suggest that RNA modifications, including m6A, can post‐transcriptionally fine‐tune the expression of key ion channel subunits (Meng and Li 2025; Chen et al. 2025; Ding et al. 2024), providing a potential link between epitranscriptomic regulation and electrical remodeling in aging atria.

In this study, we observed a significant age‐related decline in m6A levels accompanied by elevated Fto expression in murine atria. We further identified that reduced m6A modification within the coding and 3′UTR regions of Kcne1 mRNA contributes to enhanced AF susceptibility in aging mice. Importantly, restoration of m6A methylation via suppression of Fto ameliorated the arrhythmic phenotype, supporting a causal role for m6A dysregulation in age‐induced AF and highlighting the therapeutic potential of targeting the m6A pathway in treating aging‐related AF.

## 2 Results

### 2.1 Aging Is Associated With Increased AF Inducibility in Mice

We began by evaluating programmed electrical stimulation to induce AF in male mice from 15 to 24 months of age. As shown, evoked‐AF episodes were observed in aging mice from 19 months, and this grew more prominent by 24 months of age (*n* = 10/group), as measured by frequency and total duration time (Figure S1A–C). Using optical mapping (*n* = 6/group), we found there was a 1.3‐fold increase in atrial potential activation time in 19‐month‐old mice, and a 2‐fold increase in 24‐month‐old mice compared with 2‐month‐old mice (Figure S1D,E). Compared with adult mice, aging mice (19‐month‐old and 24‐month‐old) had enhanced heterogeneity of conduction, less duration of APD90 (Figure S1F,G). In addition, aging mice had a higher ratio of APD30/80 (Figure S1H), unchanged duration of APD30 and shortened duration of APD80 (Figure S1I,J), indicating that the shortening of the APD is mainly due to the repolarization phase. Whole‐cell patch clamp recordings indicated that aging mice had shorter APD90 compared to adult mice (Figure S1K,L). Moreover, there was a significant increase (1.3‐fold) in the left atrial diameter (LAD) of 19‐month‐old mice compared with 2‐month‐old adult mice, but the differences were more pronounced (1.69‐fold) in 24‐month‐old mice (Figure S1M,N). Other echocardiography parameters are presented in Table S1. Masson staining showed there was a mild increase in LA fibrosis in 19‐month‐old mice compared with 2‐month‐old mice, and more severe LA fibrosis (2.67‐fold) was observed in 24‐month‐old mice (Figure S1O,P). Guided by these insights, we focused on clarifying the mechanism for AF in mice at 19 months of age.

### 2.2 Aging Is Associated With m6A Down‐Regulation in the Atria

**FIGURE 1:**
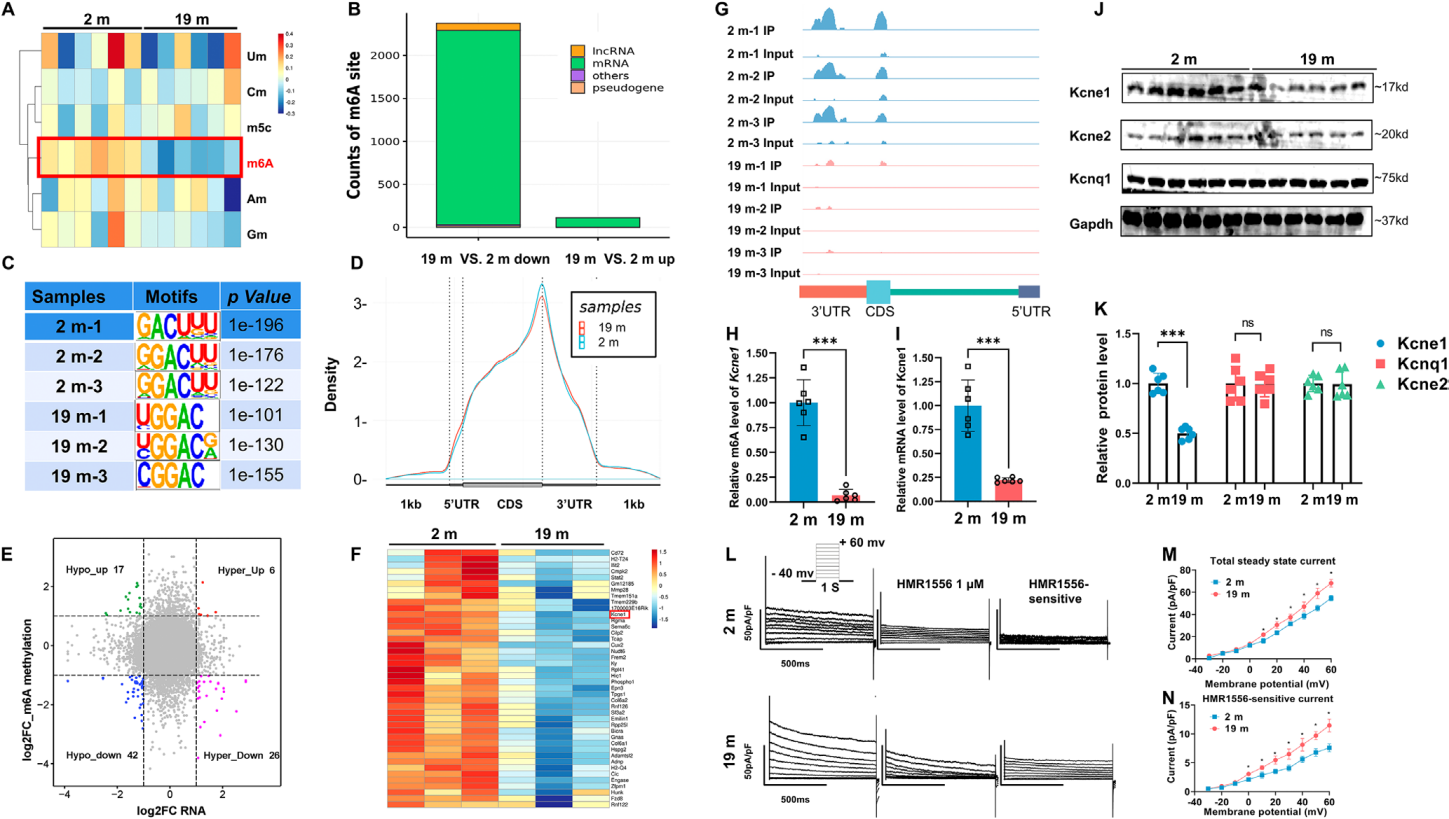
Impaired m6A modification of *Kcne1* in aging atria is associated with electrical remodeling. (A) Heat map of RNA post‐transcriptional modifications (*n* = 6/group, fold change > 2 and false discovery rate (FDR)‐adjusted *q* < 0.1). (B) Distribution of m6A peaks in different types of RNAs in atria from mice at 2 months and 19 months. (C) Predominant consensus motif GGAC was detected in atria from mice at 2 months and 19 months of age in m6A‐seq. Density distribution of m6A peaks across mRNA transcripts. (D) Regions of the 5′UTR, CDS, and 3′UTR were split into 100 segments, then percentages of m6A peaks that fall within each segment were determined. (E) Correlation between the level of gene expression and changes in m6A modification levels in atrial myocytes from mice at 2 months and 19 months of age (*n* = 3/group, fold change > 1.5 with statistical significance *p* < 0.05). (F) Heatmap of 42 genes co‐downregulated by combined transcriptomics and m6A methylomics (*n* = 3/group, fold change > 2 and false discovery rate (FDR)‐adjusted *q* < 0.1). (G) The *Kcne1* mRNA was enriched in CDS and 3′UTR in the m6A RIP‐seq data, m6A peaks were significantly reduced in 19‐month‐old mice (*n* = 3/group). (H) Statistical analysis of m6A levels of *Kcne1* mRNA (*n* = 6/group, Independent Samples *t*‐tests). (I) Statistical analysis of mRNA levels of *Kcne1* (*n* = 6/group, Independent Samples *t*‐tests). (J) Immunoblots of Kcne1, Kcne2 and Kcnq1 protein. (K) Quantification of Kcne1, Kcne2 and Kcnq1 protein levels (*n* = 6/group, Independent Samples *t*‐tests). (L) Outward current tracings recorded in atrial myocytes (Left), current tracings after exposure to the IKs/IKCNQ1 blocker HMR1556 (Middle), current tracings of HMR1556‐sensitive current (Right). (M) Current–voltage relations of outward current (8–10 myocytes/mouse, *n* = 6/group, Independent Samples *t*‐tests). (N) Current–voltage relations of HMR1556‐sensitive current (8–10 myocytes/mouse, *n* = 6/group, Independent Samples *t*‐tests). *p < 0.05; ***p < 0.001. Male mice were used for the experiment.

We performed global profiling of RNA modifications in atrial tissue samples (*n* = 6/group). As shown, among the six major mRNA modifications, m6A methylation was found to be significantly down‐regulated in 19‐month‐old mice (Figures 1A and S2A). To further investigate the variations of m6A modification in specific genes, we mapped m6A‐seq of atrial tissue from 2‐month‐old mice to 19‐month‐old mice (*n* = 3/group). Principal component analysis (PCA) showed good reproducibility of data among three biological replicates from each respective group (Figure S2B). Also, m6A‐seq analysis identified on average 9664 and 8676 m6A peaks in 2‐month and 19‐month mouse atrial tissue, respectively. Compared with 2‐month‐old mice tissues, 2362 m6A peaks were down‐regulated and 113 peaks were up‐regulated in the aging atria. The distribution patterns of m6A methylation sites are shown in Figure 1B. We found that the GGAC motif was highly enriched within m6A sites in both aging and adult groups (Figure 1C), while the peak of m6A methylation in the adult group was higher than that in the aging group (Figure 1D). Moreover, m6A peaks in the mouse atria were abundant in exon and 3′UTR regions (Figure S2C). We performed mRNA‐seq and found that, out of 16,946 evaluated genes, a total of 889 genes were differentially expressed between the two groups (*p* < 0.05, at least 1.5‐fold change) (Figure S2D). Compared with the 2‐month‐old mice, 309 genes were significantly up‐regulated, while 580 genes were down‐regulated in the aged group (Figure S2E). To define potential downstream targets associated with m6A‐dependent AF in aging mice, we identified 42 genes overlapping between 949 m6A down‐regulated genes and 580 mRNA down‐regulated genes. As shown in Figure 1E, 59 hypo‐methylated m6A genes were identified, of which the mRNA transcripts were significantly (*p* < 0.05; fold change > 1.5) down‐regulated (42; Hypo‐down) or up‐regulated (17; Hypo‐up), and 32 hyper‐methylated m6A genes were identified, of which the mRNA transcripts were significantly (*p* < 0.05; fold change > 1.5) down‐regulated (26; Hyper‐down) or up‐regulated (6; Hyper‐up) in the aged group relative to the adult group.

### 2.3 Impaired *Kcne1* m6A Modification in Aging Atria Is Associated With Electrical Remodeling

Among 42 putative candidate genes (down‐regulation in both m6A‐seq and mRNA‐seq) associated with atrial aging, *Kcne1* was the only target gene that was known to influence ion channel functions (Figure 1F). Our m6A‐seq data further confirmed that *Kcne1* mRNA featured less enrichment of m6A within its CDS and 3′UTR regions in the aging group compared to the adult group (Figure 1G). By using m6A methylated RNA‐immunoprecipitation qPCR (MeRIP‐qPCR), we verified that methylation of atrial *Kcne1* was 10‐fold lower in 19‐month‐old mice compared with 2‐month‐old mice (Figure 1H). Also, we found that mRNA levels for *Kcne1* in the atria of 19‐month‐old mice were 4‐fold lower than in 2‐month‐old mice (Figure 1I). Furthermore, Western blotting revealed that protein levels for Kcne1, but not Kcnq1 and Kcne2, were significantly decreased in the atria of 19‐month‐old mice (Figure 1J,K). IKs current densities in atrial cardiomyocytes were more intense in the aging mice than those in adult mice (Figure 1L–N).

To further characterize the function of Kcne1 in mice, we studied the effects of *Kcne1* knockdown in 2‐month‐old mice by injection of adeno‐associated virus serotype 9 (AAV9) encoding targeting sh*Kcne1*. Immunoblotting was used to validate the knockdown efficiency of *Kcne1* in atria and ventricles (Figure S3A,B). As shown, compared with control mice, the AF inducibility experiment revealed a significantly greater propensity for AF and longer AF duration in AAV9‐cTnT‐sh*Kcne1* mice (Figure S3C–E). Additionally, AAV9‐cTnT‐sh*Kcne1* mice had longer active time (3.09‐fold), enhanced dispersion of conduction (3.33‐fold), less duration of APD90 (1.79‐fold), unchanged duration of APD30, shortened duration of APD80 (1.34‐fold), and prolonged duration of APD30/80 compared to control mice (Figure S3F–L). Both total potassium and IKs currents were enhanced in AAV9‐cTnT‐sh*Kcne1* mice (Figure S3M–O). As shown, there was no difference in LAD between AAV9‐cTnT‐sh*Kcne1* mice and AAV9‐cTnT‐nc mice (Figure S3P,Q). Also, there was no significant difference in atrial fibrotic area between AAV9‐cTnT‐sh*Kcne1* mice and control mice (Figure S3R,S). Echocardiography parameters of ventricular function are presented in Table S2; no significant differences were observed in AAV9‐cTnT‐sh*Kcne1* mice compared to the control mice.

### 2.4 Aging Mice With Cardiomyocyte‐Specific *Kcne1* Overexpression Ameliorates Electrical Remodeling

To establish the function of Kcne1 in AF inducibility in aged male mice, 2‐month‐old and 18‐month‐old mice were intravenously injected with AAV9 carrying a cTnT promoter to drive *Kcne1* overexpression (AAV9‐cTnT‐*Kcne1*‐oe) (*n* = 6/group). One month after the intravenous injection of the AAV9, echocardiographic measurements were performed, followed by electrical stimulation, and then tissue harvesting for subsequent experiments. As shown, we found that overexpression of *Kcne1* reduced aging‐induced AF inducibility (Figure S4A–C). Moreover, aging‐induced traits such as prolonged active time, enhanced dispersion, shortened duration of APD90, shortened duration of APD80, and prolonged duration of increased APD30/80 in atria were also ameliorated by overexpression of *Kcne1* (Figure S4D–J). Additionally, aging‐induced enhancement in total potassium and IKs currents in atrial cardiomyocytes was reduced by overexpression of *Kcne1* (Figure S4K,L). Such observations were not made in adult mice with *Kcne1* overexpression. Furthermore, aging‐induced atrial enlargement and fibrosis were not affected by overexpression of *Kcne1* (Figure S4M,N). Based on these findings, we surmise that AF inducibility in 19‐month‐old mice is attributable to atrial electrical remodeling. Echocardiography parameters are presented in Table S3, and overexpression of *Kcne1* had no significant impact on ventricular function.

### 2.5 FTO Is Increased in the Atria of Aging Mice and Humans With AF, and Negatively Correlates With m6A Levels and KCNE1 Protein Levels

**FIGURE 2:**
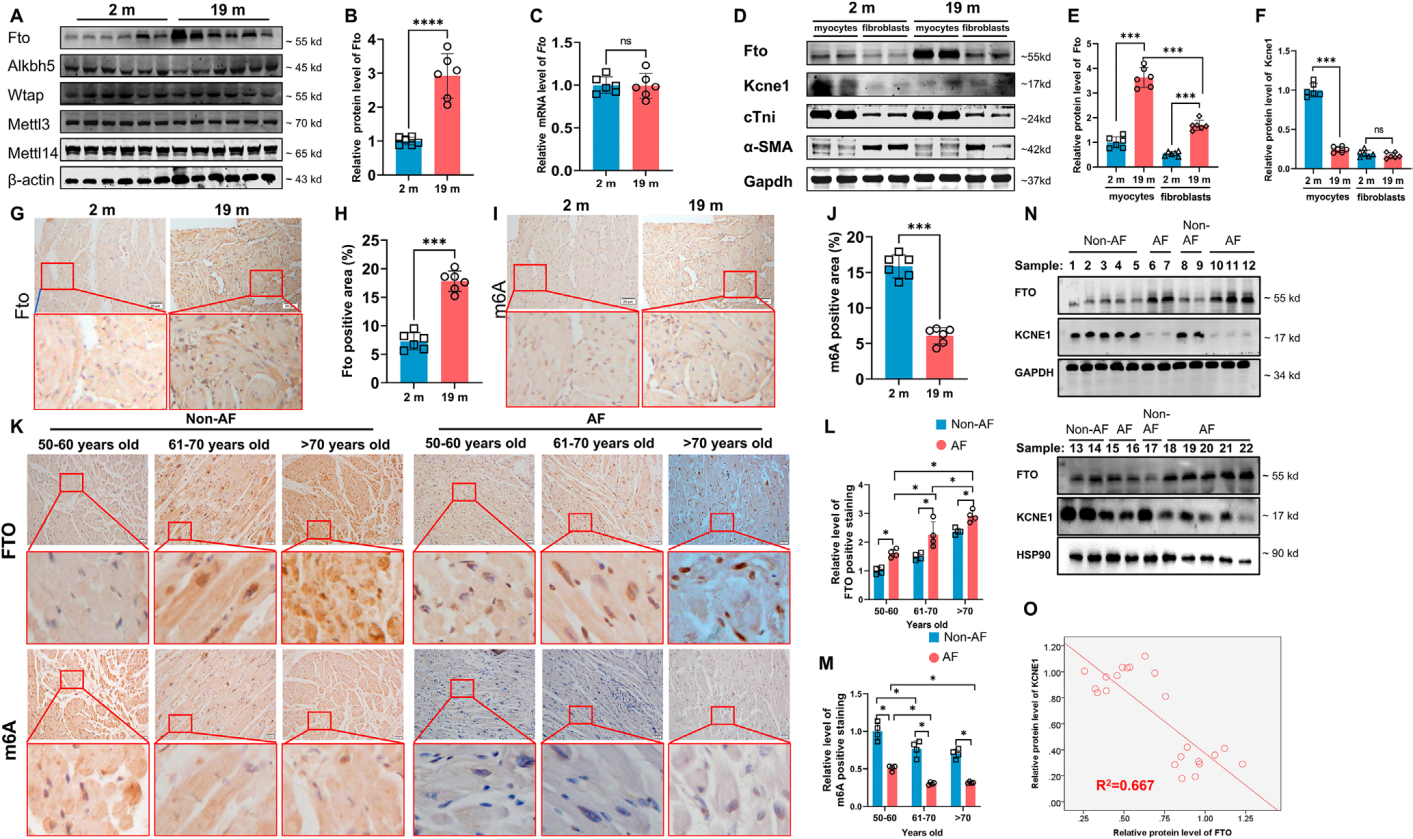
FTO levels are increased in the atria of aging mice. (A) Immunoblots of Fto, Alkbh5, Wtap, Mettl3 and Mettl14 protein. (B) Quantification of Fto protein level (*n* = 6/group, Independent Samples *t*‐tests). (C) Quantification of *Fto* mRNA level (*n* = 6/group, Independent Samples *t*‐tests). (D) Immunoblots of Fto, cTni and α‐Sma in atrial cardiomyocytes and atrial fibroblasts. (E) Quantification of Fto (*n* = 6/group, Two‐way ANOVA with Turkey's multiple tests). (F) Quantification of Kcne1 (*n* = 6/group, Two‐way ANOVA with Turkey's multiple tests). (G) Immunohistochemical Fto staining of atria from mice. (H) Quantification of m6A in G (*n* = 6/group, Independent Samples *t*‐tests). (I) Immunohistochemical m6A staining of atria from mice. (J) Quantification of m6A in I (*n* = 6/group, Independent Samples *t*‐tests). ***p < 0.001; ****p < 0.0001. (K) Immunohistochemical FTO and m6A staining of LAA from human with or without AF. (L) Quantification of FTO in K (*n* = 4/group, Two‐way ANOVA with Turkey's multiple tests). (M) Quantification of m6A in K (*n* = 4/group, Two‐way ANOVA with Turkey's multiple tests). (N) Immunoblots of FTO and KCNE1 protein of LAA from human with AF. (O) Linear correlation plot of FTO protein levels and KCNE1 protein levels in LAA from patients with or without AF (*n* = 22, Pearson Correlation Analysis). *p < 0.05. Male mice were used for the experiment.

The protein levels of the m6A demethylases Fto and Alkbh5, and the methyltransferase Mettl3, Mettl14, and Wtap in mouse atrial tissues of 2‐month‐old and 19‐month‐old male mice (*n* = 6/group) were all assessed by Western blotting (Figure 2A). As shown, protein levels for Fto were significantly increased (3‐fold) in the atria of aging mice (Figure 2B), whereas there were no differences in Mettl3, Mettl14, Wtap, and Alkbh5 between the groups. Interestingly, we found that there was no significant difference in *Fto* mRNA level between the two groups (Figure 2C), and this raised the possibility that Fto protein degradation may be blocked in aging mice. We further separated cardiomyocytes and fibroblasts in the atria and analyzed them by Western blotting to find that both atrial cardiomyocytes and fibroblasts of old mice had increased Fto protein levels; however, the differences in Fto levels in cardiomyocytes were more pronounced between 2‐month‐old and 19‐month‐old atria (Figure 2D,E). Notably, Kcne1 protein is predominantly expressed in cardiomyocytes, not fibroblasts (Figure 2F). Results from immunohistochemical staining experiments were consistent with Western blotting studies, where both showed an increase in Fto in the nuclei of atrial cardiomyocytes from 19‐month‐old mice, which corresponded to reduced m6A levels (Figure 2G–J).

We recruited 24 patients with coronary artery disease undergoing bypass surgery with preserved left ventricular function (LVEF > 50%), stratified into age‐, gender‐, and comorbidity‐matched cohorts, 12 with AF and 12 without AF. Baseline characteristics were summarized in Table S4. Left atrial appendage (LAA) samples from patients were collected; we found by immunostaining that FTO was significantly increased with tissue age. Both non‐AF patients and AF patients who had increased FTO had low levels of m6A (Figure 2K–M). AF patients had more increased FTO levels compared with non‐AF patients of similar age (Figure 2K,L). Consistent with this finding, we observed by Western blotting that FTO immunoblotted signals negatively correlated with KCNE1 levels in patients with or without AF (Figure 2N,O).

### 2.6 Validation of Aging‐Induced Electrical Remodeling and *Kcne1* m6A Demethylating in Female Mice

AF inducibility, atrial electrical conduction, intensities of total potassium and IKs currents, as well as LAD and fibrosis were tested in female mice of both 2‐month‐old and 19‐month‐old. Aging‐induced increment in AF inducibility, disturbance in atrial electrical conduction, enhancement in potassium and IKs currents, as well as atrial enlargement and fibrosis, were observed in female 19‐month‐old mice (Figure S5A–N). There were also no sex‐related differences between male mice and female mice of 2‐month‐old (Figure S5A–N). Furthermore, aging‐induced increments in Fto and reductions in Kcne1 were also verified in female aged mice, with more profound changes in Fto in female mice compared with male mice at the same age (Figure S5O–Q). The total m6A levels in the atria were comparable between male and female in adult mice, but the reductions seemed more prominent in female aged mice (Figure S5R). We also performed MeRIP‐qPCR and found that *Kcne1* m6A levels were similar between male and female mice, for both adult mice and aged mice (Figure S5S). We did not detect any significant differences in ventricular function between aged female and male mice. Echocardiography parameters are presented in Table S5.

### 2.7 Cardiomyocyte‐Specific Deletion of *Fto* Alleviates Aging‐Induced AF

**FIGURE 3:**
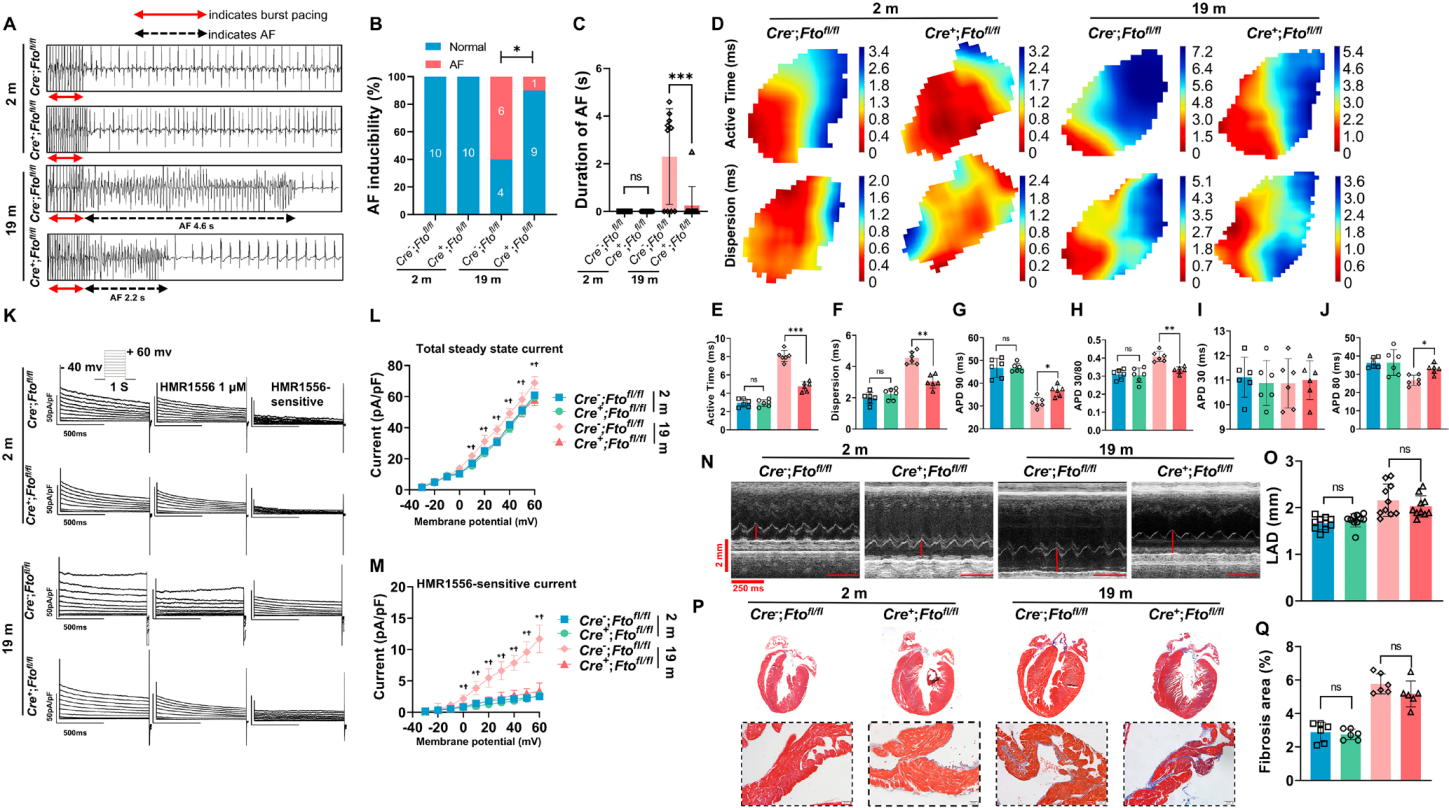
Cardiomyocyte‐specific deletion of *Fto* alleviates aging‐induced AF. (A) Representative intracardiac bipolar electrograms showing induction of AF from *Myh6‐cre* ^−^; *Fto* ^fl/fl^ mice and *Myh6‐cre* ^+^; *Fto* ^fl/fl^ mice at 2 months and at 19 months of age. (B, C) AF inducibility and total duration of AF (*n* = 10/group). (D) Representative optical maps of paced LA from *Myh6‐cre* ^−^; *Fto* ^fl/fl^ mice and *Myh6‐cre* ^+^; *Fto* ^fl/fl^ mice at 2 months and at 19 months of age. (E) Statistical analysis of active time (*n* = 6/group). (F) Statistical analysis of dispersion of conduction (*n* = 6/group). (G) Statistical analysis of APD90 (*n* = 6/group). (H) APD30/80 ratio (*n* = 6/group). (I) Outward current tracings recorded in atrial cardiomyocytes (Left), current tracings after exposure to the IKs/IKCNQ1 blocker HMR1556 (Middle), current tracings of HMR1556‐sensitive current (Right). (J) Current–voltage relations of outward current (8–10 myocytes/mouse, *n* = 6/group). (K) Current–voltage relations of HMR1556‐sensitive current (8–10 myocytes/mouse, *n* = 6/group). (L) Representative images of two‐dimensional M‐mode for the measurement of LAD at diastole, red lines indicate LAD, Time stamp: 250 ms, Vertical bar: 2 mm. (M) Quantitation of LAD (*n* = 10/group). (N) Representative images of Masson staining showing fibrotic areas in whole heart and atria from *Myh6‐cre* ^−^; *Fto* ^fl/fl^ mice and *Myh6‐cre* ^+^; *Fto* ^fl/fl^ mice at 2 months and at 19 months of age. (O) Quantitation of percentage of fibrotic area of atria (*n* = 6/group). (P), Representative images of Masson staining showing fibrotic areas in whole heart and atria from Myh6‐*cre* ^‐^; Fto*^fl/fl^* mice and Myh6‐*cre* ^+^; Fto*^fl/fl^* mice at 2 months and at 19 months of age. (Q), Quantitation of percentage of fibrotic area of atria (*n* = 6/group). Data in this figure were analyzed by two‐way ANOVA with Turkey's multiple tests. *: 19 m *Cre* ^+^; *Fto* ^fl/fl^ versus 19 m *Cre* ^−^; *Fto* ^fl/fl^ (*p* < 0.05); **: 19 m *Cre* ^+^; *Fto* ^fl/fl^ versus 19 m *Cre* ^−^; *Fto* ^fl/fl^ (*p* < 0.01); ***: 19 m *Cre* ^+^; *Fto* ^fl/fl^ versus 19 m *Cre* ^−^; *Fto* ^fl/fl^ (*p* < 0.001); †: 19 m *Cre* ^−^; *Fto* ^fl/fl^ versus 2 m *Cre* ^−^; *Fto* ^fl/fl^ (*p* < 0.05). Male mice were used for the experiment.

To establish the function of Fto in AF in aged mice, we crossed *Myh6‐cre* mice with *Fto* ^fl/fl^ mice to generate mice with cardiomyocyte‐specific *Fto* knockout mice. The *Myh6‐cre* ^+^; *Fto* ^fl/fl^ mice and *Myh6‐cre* ^−^; *Fto* ^fl/fl^ mice were kept until 19 months of age. The AF inducibility experiment revealed a significantly greater propensity for AF and longer AF duration in *Myh6‐cre* ^−^; *Fto* ^fl/fl^ mice compared with *Myh6‐cre* ^+^; *Fto* ^fl/fl^ mice at 19 months of age (*n* = 10/group) (Figure 3A–C), however, there was no such difference between the two groups of mice studied at 2 months of age. In addition, we verified that cardiomyocyte *Fto* knockout mice had shortened activation time, reduced conduction dispersion, increased APD90, reduced APD30/80 ratio (Figure 3D–H), unchanged duration of APD30, and increased duration of APD80 (Figure 3I,J) compared with control mice at 19 months of age. At the cellular level, total potassium and IKs currents recorded from atrial myocytes isolated from *Myh6‐cre* ^+^; *Fto* ^fl/fl^ mice were smaller than those recorded from *Myh6‐cre* ^−^; *Fto* ^fl/fl^ mice at 19 months of age (Figure 3K–M). Furthermore, aging‐induced atrial enlargement (Figure 3N,O) and fibrosis (Figure 3P,Q) were not reduced in the atria of *Myh6‐cre* ^+^; *Fto* ^fl/fl^ mice compared to *Myh6‐cre* ^−^; *Fto* ^fl/fl^ mice at 19 months of age. Other echocardiography parameters are presented in Table S6.

### 2.8 Adult Mice With Cardiomyocyte‐Specific *Fto* Overexpression Increases AF Susceptibility in an m6A‐Dependent Manner

**FIGURE 4:**
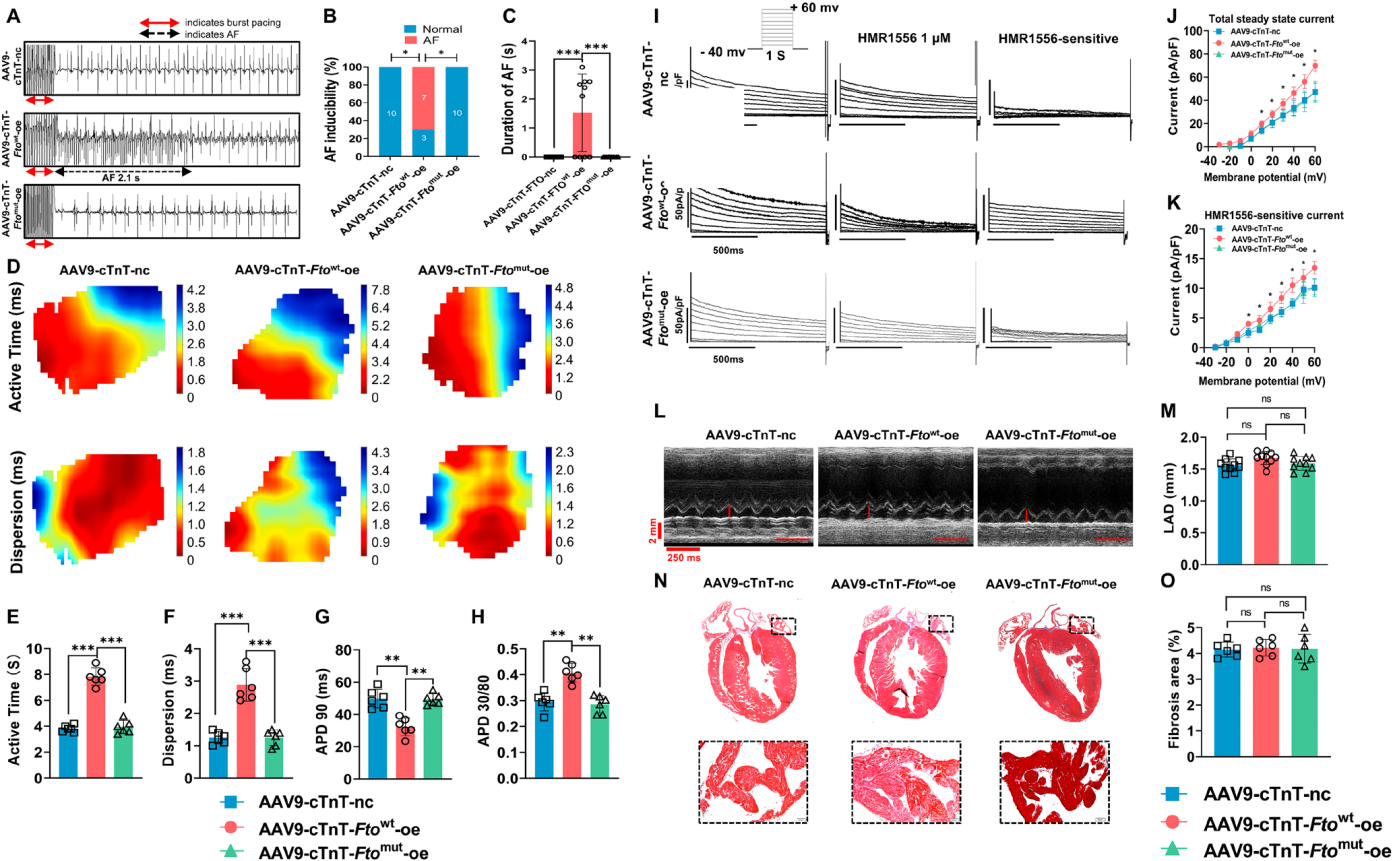
Adult mice with cardiomyocyte‐specific *Fto* overexpression increase AF susceptibility in an m6A‐dependent manner. (A) Representative intracardiac bipolar electrograms showing induction of AF from mice injected with AAV9‐cTnT‐*Fto* ^wt^‐oe, AAV9‐cTnt‐*Fto* ^mut^‐oe and AAV9‐cTnT‐nc. (B, C) AF inducibility and total duration of AF (*n* = 10/group). (D) Representative optical maps of paced LA from mice injected with AAV9‐cTnt‐*Fto* ^wt^‐oe, AAV9‐cTnt‐*Fto* ^mut^‐oe and AAV9‐cTnT‐nc. (E) Statistical analysis of active time (*n* = 6/group). (F) Statistical analysis of dispersion of conduction (*n* = 6/group). (G) Statistical analysis of APD90 (*n* = 6/group). (H) APD30/80 ratio (*n* = 6/group). (I) Outward current tracings recorded in atrial myocytes (Left), current tracings after exposure to the IKs/IKCNQ1 blocker HMR1556 (Middle), current tracings of HMR1556‐sensitive current (Right). (J) Current–voltage relations of outward current (8–10 myocytes/mouse, *n* = 6/group). (K) Current–voltage relations of HMR1556‐sensitive current (8–10 myocytes/mouse, *n* = 6/group). (L) Representative images of two‐dimensional M‐mode for the measurement of LAD at diastole from mice injected with AAV9‐cTnT‐*Fto* ^wt^‐oe, AAV9‐cTnT‐*Fto* ^mut^‐oe and AAV9‐cTnT‐nc, red lines indicate LAD, Time stamp: 250 ms, Vertical bar: 2 mm. (M) Quantitation of LAD (*n* = 10/group). (N) Representative images of Masson staining showing fibrotic areas in whole heart and atria from mice injected with AAV9‐cTnT‐*Fto* ^wt^‐oe, AAV9‐cTnt‐*Fto* ^mut^‐oe and AAV9‐cTnT‐nc. (O) Quantitation of percentage of fibrotic area (*n* = 6/group). Data in this figure were analyzed by One‐way ANOVA with Turkey's multiple tests. *: *p* < 0.05; **: *p* < 0.01; ***: *p* < 0.001; (*: vs. AAV9‐cTnT‐*Fto* ^wt^‐oe (*p* < 0.05) in J and K). Male mice were used for the experiment.

To establish the functional relationship between *Fto* expression and its demethylase function on Kcne1, 2‐month‐old male mice were intravenously injected with AAV9 to drive wild‐type *Fto* overexpression (AAV9‐cTnT‐*Fto* ^wt^‐oe), while experiments in parallel were performed in which mice were injected with AAV9 encoding a variant of *Fto* with a functional site mutation (I367F) in its demethylase (AAV9‐cTnT‐*Fto* ^mut^‐oe), which reduced such activity by 80% (Sachse et al. 2018). One month after the intravenous injection of the AAV9, echocardiographic measurements were performed, followed by electrical stimulation, and then tissue harvesting for subsequent experiments. As shown, we found that overexpression of wild‐type *Fto*, rather than its catalytically inactive mutant, was associated with a reduction in Kcne1 protein in atrial tissues (Figure S6A–C), as well as reduced total m6A level in the atria (Figure S6D). Furthermore, overexpression of wild‐type *Fto*, not mutant, increased AF inducibility (Figure 4A–C) and prolonged active time, enhanced dispersion, shortened duration of APD90, increased APD30/80 (Figure 4D–H), unchanged duration of APD30, and shortened duration of APD80 (Figure S6E,F). Total potassium and IKs currents were enhanced in atrial cardiomyocytes from AAV9‐cTnT‐*Fto* ^wt^‐oe mice, which were not observed in AAV9‐cTnT‐nc mice and AAV9‐cTnT‐*Fto* ^mut^‐oe mice (Figure 4I–K). LAD (Figure 4L,M) and fibrotic areas were not changed among three groups (Figure 4N,O). Echocardiography parameters are presented in Table S7; neither overexpression of mutant *Fto* nor wild‐type *Fto* altered ventricular function in mice.

### 2.9 Cardiomyocyte‐Specific Overexpression of *Kcne1* in Mice Mitigates the Increased Susceptibility to AF Caused by *Fto* Overexpression

**FIGURE 5:**
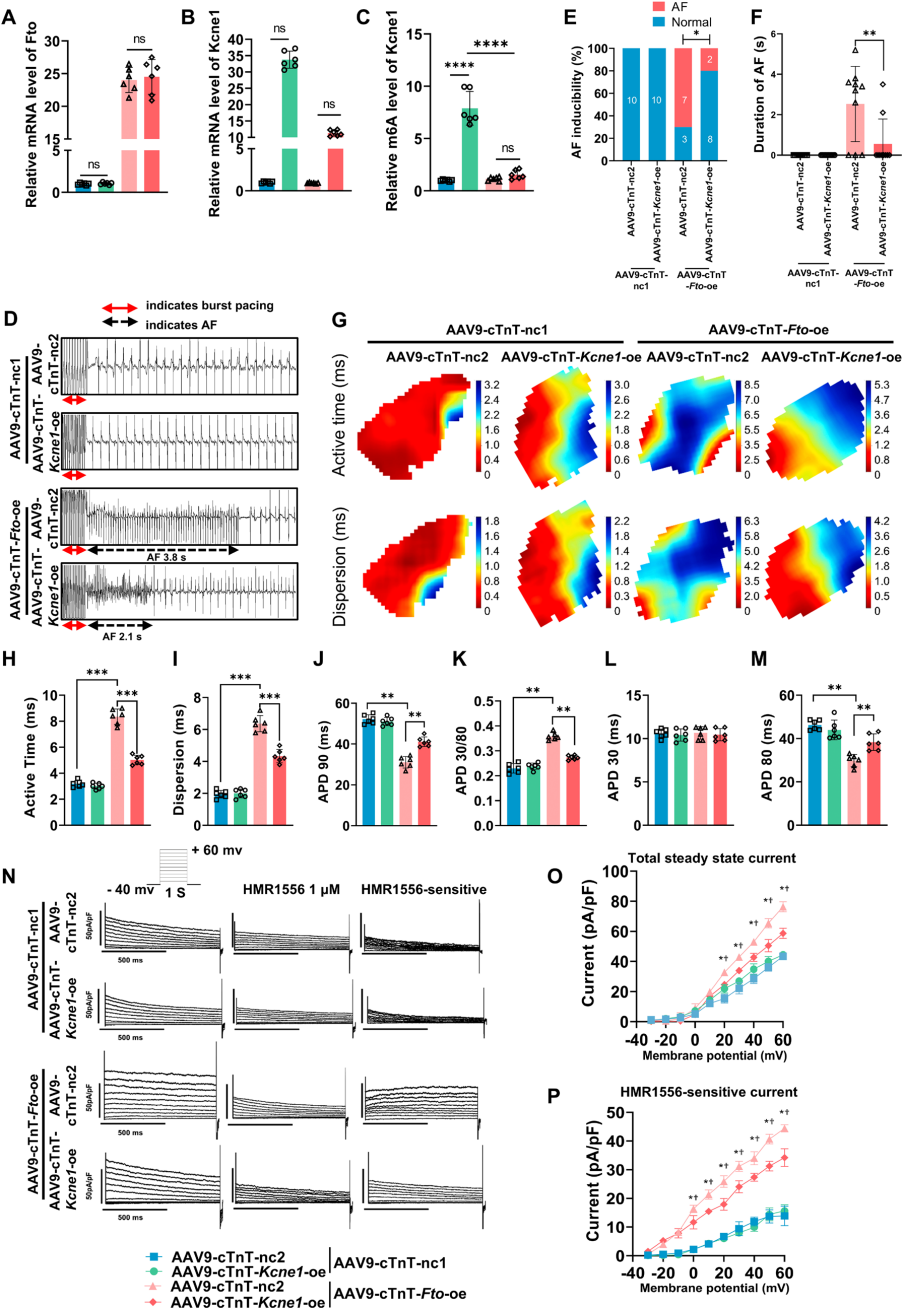
Cardiomyocyte‐specific overexpression of *Kcne1* mitigates the increased susceptibility to AF in mice caused by *Fto* overexpression. (A) Quantification of Fto mRNA level (*n* = 6/group, Two‐way ANOVA with Turkey's multiple tests). (B) Quantification of Kcne1 mRNA level (*n* = 6/group, Two‐way ANOVA with Turkey's multiple tests). (C) Statistical analysis of m6A levels of *Kcne1* mRNA (*n* = 6/group, Two‐way ANOVA with Turkey's multiple tests). (D) Representative intracardiac bipolar electrograms showing induction of AF from mice co‐injected with AAV9‐cTnT‐*Fto*‐oe and AAV9‐cTnT‐nc2, AAV9‐cTnT‐*Fto*‐oe and AAV9‐cTnT‐*Kcne1*‐oe, AAV9‐cTnT‐*Kcne1*‐oe and AAV9‐cTnT‐nc1, AAV9‐cTnT‐nc1 and AAV9‐cTnT‐nc2. (E, F) AF inducibility and total duration of AF (*n* = 6/group). (G) Representative optical maps of paced LA from mice co‐injected with AAV9‐cTnT‐*Fto*‐oe and AAV9‐cTnT‐nc2, AAV9‐cTnT‐Fto‐oe and AAV9‐cTnT‐Kcne1‐oe, AAV9‐cTnT‐Kcne1‐oe and AAV9‐cTnT‐nc1, AAV9‐cTnT‐nc1 and AAV9‐cTnT‐nc2. (H) Statistical analysis of active time (*n* = 6/group). (I) Statistical analysis of dispersion of conduction (*n* = 6/group). (J) Statistical analysis of APD90 (*n* = 6/group). (K) APD30/80 ratio (*n* = 6/group). (L) Outward current tracings recorded in atrial myocytes (Left), current tracings after exposure to the IKs/IKCNQ1 blocker HMR1556 (Middle), current tracings of HMR1556‐sensitive current (Right). (M) Current–voltage relations of outward current (8–10 myocytes/mouse, *n* = 6/group). (N) Current–voltage relations of HMR1556‐sensitive current (8–10 myocytes/mouse, *n* = 6/group). Data in this figure were analyzed by Two‐way ANOVA with Turkey's multiple tests. *: *p* < 0.05; **: *p* < 0.01; ***: *p* < 0.001; (*: AAV9‐cTnT‐*Fto*‐oe‐AAV9‐cTnT‐*Kcne1*‐oe vs. AAV9‐cTnT‐*Fto*‐oe‐AAV9‐cTnT‐nc2 (*p* < 0.05); †: AAV9‐cTnT‐nc1‐AAV9‐cTnT‐nc2 vs. AAV9‐cTnT‐*Fto*‐oe‐AAV9‐cTnT‐nc2 (*p* < 0.05) in O and P). Male mice were used for the experiment.

To verify whether *Kcne1* overexpression could rescue the increased susceptibility to AF that may be resulting from *Fto* overexpression, 2‐month‐old male mice were intravenously injected with AAV9‐cTnT‐*Fto*‐oe, with or without AAV9‐cTnT‐*Kcne1*‐oe. One month after the intravenous injection of the AAV9, echocardiographic measurements were performed, followed by electrical stimulation, and then tissue harvesting for subsequent experiments. *Fto* overexpression significantly reduced the mRNA level of *Kcne1* that is overexpressed (Figure 5A,B). Overexpression of *Fto* led to a significant reduction in the mRNA levels of overexpressed Kcne1 (Figure 5A,B). A significant decrease in m^6^A modification was observed within the CDS region of *Kcne1* in the AAV9‐cTnT‐*Fto*‐oe group compared with the control group (Figure 5C). AF could not be induced in mice with overexpression of AAV9 control and *Kcne1* (Figure 5D,E). Compared with mice with only *Fto* overexpression, increased AF inducibility was rescued in mice with overexpression of *Fto* and *Kcne1* (Figure 5D–F). Moreover, shortened active time, attenuated dispersion, prolonged duration of APD90, shortened APD30/80 (Figure 5G–K), unchanged duration of APD30, and prolonged duration of APD80 (Figure 5L,M) were found in mice which overexpressed both *Fto* and *Kcne1*. The overexpression of *Fto*‐induced enhancement of total potassium and IKs currents in atrial cardiomyocytes was also rescued by overexpression of *Kcne1* (Figure 5O,P). Other echocardiography parameters are presented in Table S8.

### 2.10 FTO Affects *KCNE1* mRNA Transcription and Translation by Decreasing *KCNE1* m6A Methylation Levels

**FIGURE 6:**
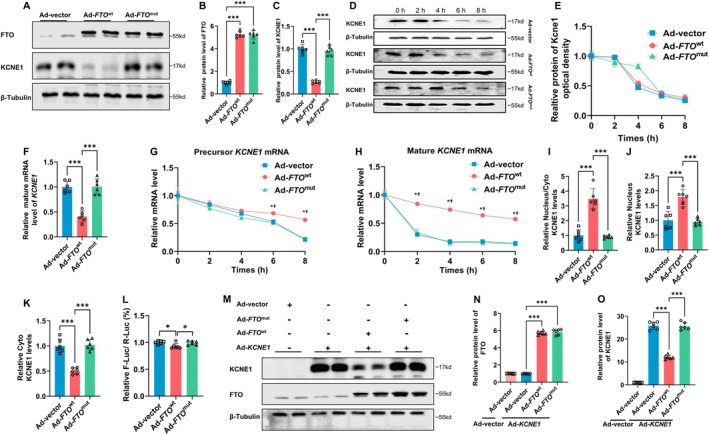
FTO affects *KCNE1* mRNA transcription and translation by decreasing *KCNE1* m6A methylation levels. (A) Representative immunoblots of FTO and KCNE1 protein of iPSC‐aCMs treated with *FTO* ^wt^ overexpression adenovirus, *FTO* ^mut^ overexpression adenovirus and blank vector adenovirus (*n* = 6/group, One‐way ANOVA with Turkey's multiple tests). (B) Quantification of FTO protein level (*n* = 6/group, One‐way ANOVA with Turkey's multiple tests). (C) Quantification of KCNE1 protein level (*n* = 6/group, One‐way ANOVA with Turkey's multiple tests). (D) Representative immunoblots of KCNE1 protein of *FTO* ^wt^ overexpression iPSC‐aCMs, *FTO* ^mut^ overexpression iPSC‐aCMs and blank vector iPSC‐aCMs treated with CHX for the indicated times. (E) Quantification of KCNE1 protein level of indicated times (*n* = 6/group, One‐way ANOVA with Turkey's multiple tests). (F) Quantification of mature *KCNE1* mRNA level of iPSC‐aCMs treated with *FTO* ^wt^ overexpression adenovirus, *FTO* ^mut^ overexpression adenovirus and blank vector adenovirus (*n* = 6/group, One‐way ANOVA with Turkey's multiple tests). (G) Quantification of precursor *KCNE1* mRNA level of indicated times (*n* = 6/group, One‐way ANOVA with Turkey's multiple tests). (H) Quantification of mature *KCNE1* mRNA level of indicated times (*n* = 6/group, One‐way ANOVA with Turkey's multiple tests). (I) The relative levels of the nuclear versus cytoplasmic *KCNE1* mRNA in *FTO* ^wt^ overexpression iPSC‐aCMs, *FTO* ^mut^ overexpression iPSC‐aCMs and blank vector iPSC‐aCMs (*n* = 6/group, One‐way ANOVA with Turkey's multiple tests). (J) The relative levels of the nuclear *KCNE1* mRNA in *FTO* ^wt^ overexpression iPSC‐aCMs, *FTO* ^mut^ overexpression iPSC‐aCMs and blank vector iPSC‐aCMs (*n* = 6/group, One‐way ANOVA with Turkey's multiple tests). (K) The relative levels of cytoplasmic *KCNE1* mRNA in *FTO* ^wt^ overexpression iPSC‐aCMs, *FTO* ^mut^ overexpression iPSC‐aCMs and blank vector iPSC‐aCMs (*n* = 6/group, One‐way ANOVA with Turkey's multiple tests). (L) *KCNE1* 3′UTR region was ligated to luciferase reporter adenovirus in iPSC‐aCMs (*n* = 6/group, One‐way ANOVA with Turkey's multiple tests). (M) Representative immunoblots of FTO and KCNE1 protein for pmirGLO‐KCNE1‐CDS adenovirus and pmirGLO‐*FTO* ^wt^‐CDS adenovirus, pmirGLO‐*KCNE1*‐CDS and pmirGLO‐*FTO* ^mut^‐CDS adenovirus, pmirGLO‐*KCNE1*‐CDS and blank vector adenovirus were co‐transfected into iPSC‐aCMs for 96 h. (N) Quantification of FTO protein level (*n* = 6/group, Two‐way ANOVA with Turkey's multiple tests). (O) Quantification of KCNE1 protein level (*n* = 6/group, Two‐way ANOVA with Turkey's multiple tests). *: *p* < 0.05; ***: *p* < 0.001; (*: Ad‐*FTO* ^wt^ vs. Ad‐vector (*p* < 0.05); †: Ad‐*FTO* ^wt^ vs. Ad‐*FTO* ^mut^ (*p* < 0.05) in G and H).

The potential mechanism for FTO‐mediated *KCNE1* m6A modification was investigated in human iPSC‐derived atrial cardiomyocytes (iPSC‐aCMs). We first confirmed the successful generation of iPSC‐aCMs by immunofluorescence staining (Figure S7A). Next, cultures of iPSC‐aCMs were infected with preparations of adenoviruses carrying empty vector (Ad‐vector), FTO (Ad‐*FTO* ^wt^), or a catalytically inactive mutant form of FTO (Ad‐*FTO* ^mut^) (Bartosovic et al. 2017). From these experiments, we found that overexpression of wild‐type *FTO*, rather than its catalytically inactive mutant, resulted in a significant decrease in KCNE1 (Figure 6A–C). Western blotting showed that there were no differences in the half‐lives of KCNE1 protein in samples of cell lysate among the three groups (Figure 6D,E), indicating that protein stability was not affected by FTO. Mature mRNA levels of *KCNE1* in Ad‐*FTO* ^wt^ cells were significantly reduced compared with the Ad‐vector group and Ad‐*FTO* ^mut^ group (Figure 6F). Notably, the half‐lives of *KCNE1* precursor mRNA (Figure 6G) and mature mRNA (Figure 6H) in Ad‐*FTO* ^wt^ cells were significantly longer than samples from the Ad‐vector group and Ad‐*FTO* ^mut^ groups, indicating that the RNA splicing and degradation of mRNA processes of *KCNE1* were delayed through a mechanism likely involving FTO‐induced *KCNE1* m6A demethylation. Moreover, by separating cytoplasmic and nuclear mRNA, we found that the nuclear/cytoplasmic ratio of *KCNE1* mRNA was only increased in Ad‐*FTO* ^wt^ cells, suggesting that reductions in m6A modification led to the retention of mRNA in the nucleus (Figure 6I–K).

The potential roles of m6A methylation on translational efficiency were studied in both the CDS and 3′UTR regions. We first constructed the pmirGLO‐*KCNE1* luciferase reporter adenovirus by ligating the nucleotide sequence for the *KCNE1* 3′UTR region to the multiple cloning site (MCS) within the vector (Figure S7B). We performed a dual luciferase assay with this vector and found that methylation in the 3′UTR region of *KCNE1* only had a mild effect on the translational efficiency (Figure 6L). In the second experiment, we infected iPSC‐aCMs with either Ad‐*FTO* ^wt^ or Ad‐*FTO* ^mut^ in concert with Ad‐*KCNE1*. We found that overexpression of wild‐type *FTO*, not the mutant form, reduced steady‐state protein levels of KCNE1 (Figure 6M–O), suggesting FTO influences KCNE1 levels likely through its m6A methylation function on the CDS of *KCNE1* mRNA to influence its translational efficiency.

### 2.11 Genetic Deletion of *Fto* or Pharmacological Inhibition of Fto Demethylase Activity Alleviates Aging‐Induced AF

We wanted to determine if knockdown of *Fto* by virus‐mediated delivery of shRNAs influenced AF, as shown in the experimental design in Figure S8A. Two‐month‐old and 19‐month‐old male mice were intravenously injected with AAV9‐cTnT‐sh*Fto* to knockdown *Fto* in cardiomyocytes. One month after the intravenous injection of the AAV9, echocardiographic measurements were performed, followed by electrical stimulation, and then tissue harvesting for subsequent experiments. Cardiomyocytes from atrial and ventricular tissues showed comparable *Fto* knockdown efficiencies, as represented by the levels of detectable Fto protein. Moreover, reduced Fto in aging cardiomyocytes was associated with changes to Kcne1 (Figure S8B–D). *Fto* knockdown led to increases in total m6A levels in the atria of both adult and aging mice (Figure S8E). As shown, aging‐induced susceptibility to AF as well as electrical remodeling was significantly reduced in mice with cardiomyocyte‐specific *Fto* knockdown, compared with the control group (Figure S8F–M). Additionally, deletion of *Fto* in cardiomyocytes also attenuated the increase in total potassium and IKs currents in aging mice (Figure S8N,O). Aging‐induced atrial enlargement (Figure S8P) and fibrosis (Figure S8Q) were not reduced in the atria of AAV9‐cTnT‐sh*Fto* mice compared with AAV9‐cTnT‐nc mice at the age of 20 months. The corresponding physiological and echocardiography parameters are shown in Table S9.

Guided by these findings, we next tested if chemical disruption of m6A methylation could modulate aging‐induced AF in our mouse model. To achieve this, we exposed 2‐month‐old and 19‐month‐old male mice to FB23, a potent and selective inhibitor of Fto, and then analyzed them after 4 weeks, as shown in Figure S9A. One month after the administration of FB23 (5 mg/kg/week, ip), echocardiographic measurements were performed, followed by electrical stimulation, and then tissue harvesting for subsequent experiments. FB23 did not have an inhibitory effect on protein levels of Fto (Figure S9B,C). Owing to the putative inhibition of demethylase activity, FB23 exposure resulted in decreased m6A levels in the atria of aging mice (Figure S9D). We further found that exposure to FB23 reduced the susceptibility to AF in aging mice, as evidenced by a reduction in the frequency of AF and a shorter duration of AF (Figure S9E,F). In addition, aging‐induced electrical remodeling in the atria, including shortened activation time, reduced conduction dispersion, increased APD90, and reduced APD30/80 ratio, unchanged duration of APD30 and prolonged duration of APD80 were all significantly affected by exposure to FB23 (Figure S9G–L). Furthermore, FB23 treatment alleviated aging‐induced increases in total potassium and IKs currents in atrial cardiomyocytes (Figure S9M,N). A significant decrease in LAD (Figure S9O) and fibrosis area (Figure S9P) were also documented in the atria of mice treated with FB23 compared with the control mice at 20 months of age. Other echocardiography parameters are shown in Table S10. Notably, FB23 treatment led to a reduction in aging‐caused body weight gain in aged mice (Table S10).

## 3 Persistence of PGE2 in Aging Atria Represses FTO Degradation by Inhibiting the Ubiquitination of FTO

Senescent cells synthesize oxylipin, which is a class of biologically active lipids that arise from the oxygenation of polyunsaturated fatty acids (PUFAs) (Cormenier et al. 2018; Dagouassat et al. 2013), and which can drive cells into senescence phenotypes (Wiley et al. 2021). Profiling of oxylipins from the atrial tissues showed that 13 species were increased while three were decreased in the atria of 19‐month‐old mice (Figure S10A). Kyoto Encyclopedia of Genes and Genomes (KEGG) analysis indicated that arachidonic acid metabolism was the most profoundly influenced (Figure S10B). Among the 13 elevated oxylipins, prostaglandin E2 (PGE2) was the most highly increased in the aged group (Figure S10C). Based on these observations, we hypothesized that persistent PGE2 in the atria may interfere with Fto degradation. To test this possibility, human iPSC‐aCMs were treated with different concentrations of PGE2. With increasing exposure to PGE2, immunoblotted levels of FTO protein concomitantly increased, while levels of KCNE1 protein decreased (Figure S10D–F). The effect of PGE2 on *FTO* at the transcriptional level was verified using qPCR, and we found there was no difference after PGE2 treatment (Figure S10G). We next performed a series of studies using the proteasome inhibitor MG132 and the autophagy inhibitor Bafilomycin A1, and found that FTO protein stability was influenced by a functional ubiquitin‐proteasome pathway, and not the autophagy pathway (Figure S11A–D). In addition, we found that ubiquitination of FTO in iPSC‐aCMs was curtailed in response to PGE2 treatment, suggesting that the presence of PGE2 repressed the ubiquitin‐mediated proteasomal degradation process of FTO in such cells (Figure S10H). Guided by this insight, we investigated the levels of Fto ubiquitination in the atria of mice to find that such levels in 19‐month‐old mice were lower than those in 2‐month‐old mice (Figure S10I).

## 4 Discussion

Although the role of m6A modification in a variety of biological processes has been well documented, its function and molecular regulation in cardiomyocytes, especially in the context of aging cardiomyocytes, are poorly understood. In this study, we have profiled and compared the m6A epitranscriptomes in the atria and demonstrated that Fto‐induced loss of m6A modification in aging mouse atria underlies AF susceptibility. Notably, we have made several novel findings in this study. Firstly, we find that the CDS and 3′UTR regions of *Kcne1* are m6A demethylated in aging mouse atria. Secondly, aging mice with cardiomyocyte‐specific *Fto* knockout exhibit up‐regulation of *Kcne1* at both the mRNA and protein levels, impair aging‐induced shortening of APD90, and modify susceptibility to AF. Overexpression of wild‐type *Fto* into mouse cardiomyocytes, rather than its catalytically inactive mutant, reduces atrial Kcne1 protein levels, causes shortening of APD90, enhances IKs current, and increases AF inducibility. Thirdly, FTO‐mediated *KCNE1* m6A demethylation represses *KCNE1* precursor mRNA splicing, nuclear export, and translational efficiency of *KCNE1* mRNA. Furthermore, we confirm that FTO protein levels in LAA samples from AF patients increase with advancing age, and this correlates with reduced levels of KCNE1.

We observed a reduction in m6A and protein levels of Kcne1 in atria from 19‐month‐old mice, which was associated with enhanced AF inducibility. KCNE1 expression is not sufficient for cardiac cells to elicit current potentials, as it requires an association with KCNQ1 to form a channel complex that generates the potassium current IKs. IKs was documented to increase in LA and right atrial myocytes from patients with persistent AF (Caballero et al. 2010). Moreover, mutations of either *KCNQ1* or *KCNE1* caused familial AF by increasing the repolarizing outward IKs current and shortening APD in atria (Chen et al. 2003; Olesen et al. 2012). Several forms of *KCNE1* polymorphism were also reported to be associated with AF (Han et al. 2014; Jiang et al. 2017; Voudris et al. 2014). Supporting our findings, previous animal studies have demonstrated that *Kcne1* knockout mice displayed frequent spontaneous episodes of AF by enhancing the amplitude of the repolarizing outward IKs current in atrial myocytes, but with structurally normal hearts (Temple et al. 2005). The protein level of Kcne1 was decreased after 1 week of right atrial tachypacing in rabbits (Jia et al. 2013). Silencing of *Kcne1* induced a greater increase in IKs in the atrial cardiomyocytes of rabbits and enhanced the inducibility and duration of AF (Jia et al. 2013). We further confirmed that cardiomyocyte‐specific *Kcne1* knockdown by AAV9 promoted IKs current and increased AF inducibility in mice, without affecting atrial structure. Additionally, aging‐induced enhancement of IKs currents in atrial cardiomyocytes, as well as AF inducibility, was markedly reversed by overexpression of *Kcne1*. Importantly, overexpression of *Kcne1* in the heart had no effect on aging‐induced LA expansion and fibrosis, indicating that AF inducibility in 19‐month‐old mice was attributable to the effects arising from reductions in m6A Kcne1‐mediated electrical remodeling in the atria.

Emerging evidence suggests that loss of m6A modifications contributes to the aging process and aging‐related diseases (Shafik et al. 2021; McMahon et al. 2021; Sun et al. 2022). We are the first to generate m6A epitranscriptomes in mouse atria during physiological aging and identified that enhanced Fto in atria influences aging‐associated AF. By further isolating cardiomyocytes and fibroblasts from mouse atria, we found that Fto was increased in both the cardiomyocytes and fibroblasts of aging mice, with more pronounced changes in cardiomyocytes. To more precisely define the role of Fto in aging cardiomyocytes, we created cardiomyocyte‐specific *Fto* knockout mice, as well as the overexpression of wild‐type or mutant forms of *Fto* in cardiomyocytes by AAV9 transduction. We found, firstly, that Fto protein was expressed at relatively low levels in the atria of adult mice, and was elevated in aging atria. Secondly, when Fto was selectively deleted in cardiomyocytes, 19‐month‐old mice demonstrated reduced AF susceptibility due to enhanced IKs current and prolonged APD through modulation of *Kcne1*. Thirdly, overexpression of wild‐type *Fto* in cardiomyocytes of adult mice can mimic aging‐induced electrical remodeling, and such phenotypes could not be observed in parallel experiments in which a catalytically inactive mutant form was overexpressed. Lastly, overexpression of wild‐type *Fto* in cardiomyocytes induced elevation of AF susceptibility, and IKs current could be rescued by overexpression of *Kcne1*. Collectively, aging‐induced electrical remodeling, including more intense IKs current and shortened APD, was predominated by enhanced Fto‐induced *Kcne1* m6A demethylation.

In the present study, aging induced Fto increasing in atria is only detected at the protein level. Previous and our studies have implicated the ubiquitin‐proteasome system in controlling the degradation of FTO protein (Zhu et al. 2018). FTO undergoes ubiquitination through an evolutionarily conserved Lys‐216 residue, which directs FTO toward proteasomal degradation (Zhu et al. 2018). Several studies demonstrated that senescent cells synthesize oxylipins, including prostaglandins, which are a class of biologically active lipids that arise from the oxygenation of PUFAs (Cormenier et al. 2018). Wiley et al. (2021) further confirmed that prostaglandins drive IMR‐90 fibroblasts into senescence phenotypes. Consistent with these previous findings, our investigation of oxylipin profiles showed that PGE2 was elevated in the atria of aging mice. We further found that PGE2 inhibited the ubiquitination of FTO and resulted in the accumulation of FTO within cardiomyocytes. PGE2 exerts various actions through each of its receptor subtypes (EP1, EP2, EP3, and EP4), which are differentially expressed and function differently in the heart (Sugimoto and Narumiya 2007). A previous study showed that the PGE2 prevented ubiquitin‐mediated degradation via activation of EP3 (Tao et al. 2022). Further studies will be important to clarify the receptor and underlying mechanisms through which PGE2 mediates Fto ubiquitin inhibition, and perhaps to even develop specific EP receptor antagonists to treat heart conditions, rather than to directly block PGE2 synthesis.

Several studies prior to the discovery of m6A indicated that *FTO* polymorphism was strongly associated with obesity in humans (Zhao et al. 2019; Meyre et al. 2010; Dina et al. 2007). It is not clear whether such single‐nucleotide polymorphisms in *FTO* affect its demethylase functions or some other property of this protein. Animal studies further support the role of Fto in regulating food intake, fat metabolism, and body weight. Overexpression of *Fto* led to reduced fat metabolism and obesity in mice (Church et al. 2010), coinciding with the phenotypes observed in aging mice in our study, while deficiency of *Fto* in mice protected against obesity (Fischer et al. 2009). FTO controls exonic splicing of adipogenic regulatory factor RUNX1 partner transcriptional co‐repressor 1 by regulating m6A levels around splice sites, thereby leading to modulation of adipogenesis (Zhao et al. 2014). This indicates that *FTO* polymorphisms related to obesity are likely via its demethylase activity (Zhao et al. 2014). In the present study, cardiomyocyte *Fto* deletion mice by AAV9 reserved aging‐induced AF inducibility increasing, without body weight loss. FB23 directly binds to Fto and selectively inhibits its m6A demethylase activity. Treatment with the Fto inhibitor FB23 reduced the incidence of AF inducibility in aged mice. Due to the role of Fto in regulating fat metabolism and obesity, FB23 treatment was also associated with the reduction in aging‐induced body weight gain. The protective role of FB23 in aging mice may be from both atria and weight loss. In a Mendelian randomization study of > 50,000 individuals of European ancestry, *FTO* polymorphism associated with obesity was significantly associated with an increased risk of incident AF (Chatterjee et al. 2017). *FTO* polymorphism may directly cause AF in humans, rather than through a secondary mechanism involving body weight control. Our findings are consistent with such a concept.

Our study has two main limitations. First, the expression of *Fto* mRNA was low in the heart under physiological conditions (Zhang et al. 2019) and may have limited physiological roles. Previous reports indicate that cardiomyocyte‐specific *Fto* knockout exacerbated contractile dysfunction under stress (Mathiyalagan et al. 2019), suggesting a protective role in ventricular function during disease. Similar findings were observed by Berulava et al. (2020), who reported that cardiomyocyte‐specific *Fto* knockout mice featured a more severe contractile dysfunction when subjected to transverse aortic constriction (TAC). These findings reinforced the notion that Fto contributed to the preservation of heart contractile function in diseased states. In our aged model, the observed preservation of ventricular contractility might be related to age‐related Fto upregulation. However, ventricular *Fto* knockdown did not significantly affect ejection fraction, possibly due to functional compensation by other demethylases such as Alkbh5, which could also regulate contraction‐related mRNAs. Nonetheless, we cannot rule out the possibility of adverse ventricular effects following Fto intervention. Second, although both cardiomyocyte‐specific and systemic Fto inhibition effectively improved aging‐related atrial conduction and reduced AF susceptibility, with less atrial fibrosis and dilatation in aging mice, the effects of systemic Fto inhibition on atrial fibrosis and dilatation remained incompletely understood. Our results suggested that although Fto upregulation in cardiac fibroblasts of aging atria was less robust than in cardiomyocytes, it may still contribute to fibroblast proliferation or activation. While decreased m6A modification is a hallmark of aging across multiple organs (e.g., liver and skeletal muscle) (Wu et al. 2023), and systemic Fto inhibition may confer protective benefits in these tissues, comprehensive analyses of off‐target organ effects were not performed.

In summary, we have characterized the role of the RNA demethylase Fto in aging atria. We find that aging‐induced elevation of Fto by PGE2 represses m6A methylation of *Kcne1* in the atria which, in turn, leads to the reduction of Kcne1 mRNA and protein. Reduced Kcne1 enhances IKs currents and thereby shortens APD in atrial cardiomyocytes and increases AF inducibility (see Graphical Summary). We further confirmed the relationship of FTO and KCNE1 in human LAA samples, and their regulatory mechanism in human iPSC‐aCMs, raising the possibility that FTO could be a promising therapeutic target for aging‐induced AF in human.

## 5 Materials and Methods

Detailed methods and materials are provided in the Supporting Information materials.

## Author Contributions

Conceptualization: Yang Liu and Xiaomeng Yin. Methodology: Ruopeng Tan, Mengyang Yuan, Lin Wang, Xinyu Yang, Yuanjun Sun, and Genlong Xue. Investigation: Yang Liu, Ruopeng Tan, Yuanjun Sun, and Guiwen Xu. Visualization: Ruopeng Tan. Supervision: Yang Liu, Xiaomeng Yin. Writing original draft: Yang Liu and Ruopeng Tan. Review and editing: Yang Liu and Xiaomeng Yin.

## Ethics Statement

The clinical study was approved by the Ethics Committee of the First Affiliated Hospital of Dalian Medical University; all participants provided written informed consent, and the study conformed to the principles outlined in the Declaration of Helsinki. The approval number is PJ‐KS‐KY‐2021‐229. All animal experimental procedures in this study were approved by the Animal Care and Use Committee of Dalian Medical University (AEE22063).

## Conflicts of Interest

The authors declare no conflicts of interest.

## Supporting information


**Appendix S1:** acel70263‐sup‐0001‐AppendixS1.pdf.

## Data Availability

The data that support the findings of this study are available on request from the corresponding author.
