# Supplementary material for: The m6A Demethylase Fto Enhances Susceptibility to Atrial Fibrillation by Demethylating Kcne1 in Aging Mice
Source: Aging Cell. 2025 Oct 12;24(12):e70263. doi: 10.1111/acel.70263 (PMC12686553; doi:10.1111/acel.70263)
Supplement: Supplementary file 1 — Appendix S1: acel70263‐sup‐0001‐AppendixS1.pdf. [file ACEL-24-e70263-s001.pdf]

Experimental animals

All animal procedures and the necessary numbers of animals for the experiments were approved by the Institutional Animal Care and Use Committee at Dalian Medical University (AEE22063). The animal care and experiments were also in accordance with the guidelines provided by the NIH. At the euthanasia procedure, the mice were anesthetized by intraperitoneal administration of 150 mg/kg pentobarbital sodium.

Wild type mice (C57BL/6J, male) at 2, 15 to 19, and 24 months of age were used respectively. Female mice at the age of 2 months and 19 months were used to validate the traits observed from the atria of aged male mice. Mice with cardiomyocyte-specific *Fto* deletion (*Myh6-cre*$^{+}$; *Fto*$^{fl/fl}$) were generated on a C57BL/6J background (NM-CKO-190005, Shanghai model organisms, China) and collected at 2 months and 19 months of age for analysis (male and female). To knock down the expression of *Kcne1* and *Fto*, mice were intravenously injected with adeno-associated virus serotype 9 carrying a cardiac-specific troponin T promoter that drives *Kcne1* (AAV9-cTnT-sh*Kcne1*) or *Fto* (AAV9-cTnT-sh*Fto*) knockdown by RNA interference (RNAi), with control experiments conducted using a corresponding empty AAV9 virus control (AAV9-cTnT-nc1 and AAV9-cTnT-nc2). Virus titers of 0.5 × 1011 vector genomes per gram of body weight (vg/g) were delivered as single dosages for each type of virus. To overexpress wild-type Fto (*Fto*^{wt}) or Fto harbouring a point mutation, which abolishes its demethylase catalytic activity (*Fto*^{mut}), into cardiomyocytes, AAV9 injections of each expression construct was carried out. The Fto demethylase activity inhibitor FB23 (5 mg/kg/week) (Cat# HY-137187, MCE, USA) was administered for 4 weeks in 2-month-old and 19-month-old mice before analysis.

Clinical sample collection

24 patients with coronary artery disease undergoing bypass surgery with preserved left ventricular function (LVEF >50%), stratified into age-, gender-, and comorbidity-matched cohorts, 12 with AF and 12 without AF. Left atrial appendages (LAA) from patients were collected during bypass surgery and then fixed in 4% paraformaldehyde. After embedding in paraffin, 4 μm sections of samples were floated onto glass slides, dried and then processed for HE staining, Masson staining and immunohistochemistry (IHC). The clinical study was approved by the Ethics Committee of the First Affiliated Hospital of Dalian Medical University, all participants provided written informed consent,

and the study conformed to the principles outlined in the Declaration of Helsinki. The approval number is PJ-KS-KY-2021-229.

Induction of AF

AF induction in mice was performed under anesthesia. Following a 10-min equilibration period after anesthesia, and then an electrophysiology catheter (1.1F, Cat# FTS-1113A-0518, Transonic Science, Canada) was inserted into the right atrium via the right jugular vein. Atrial pacing was achieved using an isolated stimulator (Cat# GY-6328B, Huanan Medical Science and Technology, China). A data acquisition unit was used to deliver electrical impulses and record intracardiac electrograms as described previously. Stimulation was delivered at a voltage magnitude of 3, 5 and 8, at 11 different frequencies (40, 38, 36, 34, 32, 30, 28, 26, 24, 22, and 20 Hz, receptively) for 5 s duration each. AF was defined as reproducible episodes of rapid, irregular and continuous atrial rhythm lasting for more than 2 seconds. AF duration was calculated as the total period of time during AF episode in each individual mouse. AF frequency was expressed as a ratio of pacing-triggered AF episodes/33 pacing bursts in each individual mouse.

Optical mapping of *ex vivo* heart preparations and arrhythmia induction protocol

Mice were sacrificed after deep anesthesia. The hearts were isolated and Langendorff perfused with Tyrode's solution (NaCl 128.2 mM, $CaCl_2•2H_2O$ 1.3 mM, KCl 4.7 mM, $MgCl_2•6H_2O$ 1.85 mM, $NaH_2PO_4•2H_2O$ 1.19 mM, Na_2CO_3 20 mM, and glucose 11.1 mM; pH 7.35) at 37°C. When Langendorff-perfused hearts reached steady state, contraction artefacts were minimized using blebbistatin (10 µM, Cat# ab285433, Abcam, UK). The hearts were stained with RH237 (10 µM, Cat# sc-499456, Santa Cruz, California, USA) for membrane voltage (Vm) mapping that lasted 10 min. The hearts were excited at 710 nm wavelength for Vm using a one-wavelength light emitting device (Cat# LEDC-2001, MappingLab Ltd, UK). The raw spatial resolution was 1 ms/frame and 100 × 100 pixels with a spatial resolution of 0.35 × 0.35 mm^2 per pixel.

For programmed electrical stimulation (PES) induced arrhythmia in optical mapping, stimuli were generated by an isolated constant voltage/current stimulator (Cat# VCS3001, MappingLab Ltd, UK). They were delivered with a platinum electrode into the root of the aorta near to the sinus node for pacing at an amplitude that was thrice the diastolic current threshold, and with a 2 ms pulse width.

The optical mapping was performed in conjunction with an “alternans mapping” protocol. The atria were initially paced at PES (200, 150, 120, and 100 ms) with a duration of 3 s to record Vm Pacing was performed with S1–S2 using a basic PES of 100 ms and with S2 decreased by 2 ms until the ventricular effective refractory period was reached.

For the analysis of optical mapping signals, data were processed semi-automatically using OMAPScope 5.0 software (MappingLab Ltd, UK). In this experimental configuration, stable records of the voltage (Vm) signal can be obtained for >2 h in preliminary experiments to assess the stability of the system.

Echocardiography for mice

Mice were lightly anesthetized with isoflurane (1.5%, Cat# 792632, Sigma-Aldrich, USA) and checked for their spontaneous breathing. A VEVO 1100 high-resolution imaging system (VisualSonics, Canada) equipped with a 30 MHz RMV (real-time microscopic imaging) scanning head was used to obtain two-dimensional guided M-echoes (30 MHz) on short- and long-axis views. The chest was shaved, acoustic coupling gel was applied, and a warming pad was used to maintain normothermia. Mice were imaged in a shallow left lateral decubitus position. Left atrial dimension (LAD) at end-diastole was analyzed. For left ventricle (LV) measurements, dimensions at end-diastole and end-systole, anterior wall and posterior wall thickness of end-diastole and end-systole were analyzed. Ejection fraction (EF%) and fractional shortening (FS%) were calculated.

Isolation of cardiomyocytes and fibroblasts from mouse atria

Through enzymatic digestion and Langendorff retrograde perfusion, mouse atrial myocytes were extracted, as previously described. To avoid coagulation, mice were treated with heparin (100 USP units IP) 15 minutes before being sacrificed. Mice were anesthetized through 2% isoflurane inhalation before they were sacrificed by cervical dislocation. Using a modified Langendorff apparatus, the hearts were quickly removed and retrogradely perfused through the aorta at a constant flow rate (2.0 ± 0.1 mL/min) and temperature (37 ± 1°C). The hearts were perfused with the following solutions: Tyrode's solution buffered with HEPES (in mM: 130 NaCl, 5.4 KCl, 1 CaCl2, 1 MgCl2, 0.33 Na2HPO4, 10 HEPES, 5.5 glucose, pH adjusted to 7.4 with NaOH) for 5 min, Tyrode's solution without Ca2+ for 10 minutes, and 25-30 minutes with digest solution consisting of Ca2+-free Tyrode's

solution with 0.03 mM $CaCl_2$, 20 mM taurine, 0.1% bovine serum albumin (BSA) and 73.7 U/mL type II collagenase (Worthington Biochemicals, New Jersey, USA) with Kraft-Bruhe (KB) solution (units: mM: 100 K^+-glutamate, 10 K^+-aspartate, 25 KCl, 10 KH_2PO_4, 2 $MgSO_4$, 20 taurine, 5 sarcosine, 0. 5 EGTA, 5 HEPES, 20 glucose, 0.1% BSA, pH adjusted to 7.2 with KOH). The left atrium was then isolated, minced, and triturated until individual atrial myocytes were obtained. The cells were then stored at 4°C and used within one to six hours. For experiments, rod-shaped myocytes were selected.

Whole-cell patch clamp

A perfusion chamber of an inverted microscope was utilized to keep aliquots of left atrial cardiomyocytes at 37°C in extracellular fluid as the following solution (130 mM NaCl, 10 mM HEPES, 5 mM KCl, 1 mM $MgCl_2*6H_2O$, 1 mM $CaCl_2*H_2O$, 12.5 mM D-Glucose; pH adjusted to 7.4 with 5 M NaOH) while supplying cells with oxygen-rich fluids at a rate of 2 mL/min. Prior to the beginning of the solution flow, cells were left to stand for 10 minutes to enable them to sink to the bottom of the chamber. Voltage-clamp current measurements were made using a whole-cell arrangement with an Axopatch 700A patch-clamp amplifier, 1550A Digital to Analog Converter and pCLAMP 10.6 software (Molecular Devices, USA). Pipettes were pulled from borosilicate glass (Cat# P-97, Sutter, USA) and had resistances between 1 to 2 MΩ when filled with the pipette solutions. Recordings were acquired at a sampling rate of 4 kHz. All currents are expressed as current densities (pA/pF). Voltages were not corrected for liquid junction potentials as they were calculated to be less than 4 mV. All experiments were carried out at 37°C. Slowly activated delayed rectifier potassium currents (IKs) were recorded in whole-cell configuration using pipettes filled with the following solution (65 mM KCl, 75 mM KF, 10 mM HEPES, 5 mM EGTA, 2 mM $MgCl_2*6H_2O$, 200 μg/ml Amphotericin B; pH adjusted to 7.3-7.4 with 1 M KOH). Cells were held at -80 mV and pulsed to +60 mV for 5 seconds (IKs) or 1 second (IKCNQ1). Interpulse intervals were ≥15 seconds. HMR 1556 (Cat# HY-106369, MCE, USA) was added to the bath solution at a final concentration of 1μM and incubated for 5 min before the non-IKs current was tested.

Maintenance and differentiation of human iPSC-derived atrial cardiomyocytes

Human iPSCs were obtained from Help Therapeutics (Nanjing, China). The protocol of iPSC-derived

atrial cardiomyocytes differentiation was described previously. Briefly, human iPSCs were seeded onto matrigel-coated 6-well plates. Cells were cultured and expanded to 85% cell confluence and then treated for 2 days with 6 μM CHIR99021 (Cat# HY-10182, MCE, USA) in RPMI 1640 culture medium (Cat# 11875119, Gibco, USA) and B-27 Minus Insulin (Cat# A1895601, Gibco, USA) to induce Wnt signaling. On day 5-6, 2 μM retinoic acid (Cat# HY-B0351, MCE, USA) was applied to induce atrial cardiomyocyte differentiation. From day 7 onwards, cells were placed and cultured in RPMI 1640 culture medium and B-27 supplement with insulin (Cat# 17504044, Gibco, USA) until beating was observed by microscopy.

Identification of human iPSC-aCMs

The iPSC-aCMs were identified via immunofluorescence staining of cardiac-specific marker cTnI (1:100, Cat# MA1-20112, Invitrogen, USA) and the atrial-specific marker MLC2a (1:100, Cat# PA5-30789, Invitrogen, USA) and NR2F2 (1:100, Cat# PA5-46850, Invitrogen, USA) in iPSC-aCMs.

Co-Immuno-precipitation

For co-immuno-precipitation, FTO antibody (50 ug, Cat# sc-271713, Santa Cruz Biotechnology, USA) was used to incubate with co-immunoprecipitation buffer (50 mM Tris-Cl pH 7.5, 15 mM EDTA, 100 mM NaCl, 0.1% Triton X-100 and protease inhibitor cocktail obtained from Sigma at 4°C in duplicate. Ig G antibody was added to the other vial (negative control). After that, a slurry of protein-G agarose beads (50 ul) was added to both the vials and these were kept shaking overnight at 4°C. Next day, beads were precipitated by centrifuging at 1200 g for 5 min at 4°C and washed thrice with the co-immuno-precipitation buffer. Subsequently, sample buffer was added to beads and the proteins extracted by boiling. Proteins were analyzed by western blot.

Protein stability

iPSC-aCMs were infected with adenoviruses driving *FTO*$^{(wt)}$ overexpression or *FTO*$^{(mut)}$ overexpression or empty vector. After 48 h, cells were treated with cycloheximide at 100 μg/ml (CHX, Cat# HY-12320, MCE, USA) for 0, 2, 4, 6, and 8 h. Steady state levels of immunoblotted KCNE1 protein in the samples were measured by western blot.

RNA extraction and Real-time PCR for gene expression analysis

Total RNA was extracted from atrial tissues or cells using TRIzol (Cat# IS10007, Thermo Fisher, USA) according to the manufacturer's instructions. One microgram of total RNA was reverse transcribed, following which real-time PCR were performed as follows: one cycle of 5 min at 95℃, followed by 30 cycles of 30 sec at 95℃, 30 sec at 54℃, and 15 sec at 72℃. For quantitative PCR, *Actb* was used as the endogenous control for cytoplasmic RNA while 18S RNA was chosen as the endogenous control for nuclear RNA. The sequences of gene specific primers used are listed in supplemental table 1.

Nuclear/cytoplasmic mRNAs separation

Cytoplasmic & Nuclear RNA Purification Kit (Cat# 21000, Norgen Biotek, Canada) was used to isolate and purify cytoplasmic and nuclear RNA extracted from atrial tissues or cells according to the manufacturer's instructions. Briefly, add 200µL of ice-cold Lysis Buffer J directly to lyse cells, spin lysate for 3 minutes at maximum speed, transfer the supernatant containing cytoplasmic RNA to another RNase-free tube. Add add 200µL of Buffer SK to the supernatant, Add 200µL of 100% ethanol to the mixture, centrifuge for 1 minute at 6000 rpm. Add 200µL of Buffer SK to the pellet, binding Nuclear RNA to Column.Apply 400µL of Wash Solution A to the column and centrifuge for 1 minute. Discard the flowthrough. Add 50µL of Elution Buffer E to the column, spin the column at 14,000 RPM for 1 additional minute. The purified RNA samples were used for subsequent Real-time PCR.

m6A RNA Methylation Assay

Total RNA was extracted using TRIzol (Cat# IS10007, Thermo Fisher, USA) according to the manufacturer's instructions. The m6A RNA Methylation Assay Kit (Cat# ab185912, Abcam, United States) was used to assess the global m6A modification levels of the mRNA. Briefly, 200 ng of poly(A) purified RNA was added to each well, following which the relevant antibody was added to each well individually at the appropriate dilution. The OD450 of each well was measured. m6A modification levels were quantified from the standard curve and calculated.

MeRIP-qPCR

Samples of RNA from tissues and cells extracted with TRIzol (Cat# IS10007, Thermo Fisher, USA) were used for MeRIP-qPCR experiments. A CUT&RUN m6A RNA Enrichment (MeRIP) Kit (Cat# P-9018, Epigentek, USA) was used for measuring m6A methylated RNA. Briefly, after retaining 50 ng of total RNA as input, the remaining RNA (2 μg) from each sample was used for m6A-immunoprecipitation with m6A antibody in 500 μL of IP buffer to obtain a m6A pull-down product. Next, m6A RNA was immunoprecipitated with and eluted twice with elution buffer. m6A IP RNAs were recovered by ethanol precipitation, and 2 ng of the total RNA and m6A IP RNA was used as templates, respectively, in qPCR experiments, as described above.

RNA stability

iPSC-aCMs were infected with adenoviruses driving *FTO*$^{(wt)}$ overexpression or *FTO*$^{(mut)}$ overexpression or empty vector. After 48 h, actinomycin D (Act-D, Cat# HY-17559, MCE, USA) at 5 μg/ml was added to cells. After incubation at 0, 2, 4, 6, and 8 h, cells were collected, and then performed Real-time PCR. The half-lives (t1/2) for precursor and mature *KCNE1* mRNAs were calculated using ln2/slope and *ACTB* was used for normalization.

Luciferase reporter assays

Cells for luciferase assays were prepared using reporter lysis buffer (Cat #MA0518, Meilunbio, China) and luciferase assay reagent according to the manufacturer's instructions. Briefly, iPSC-aCMs were infected with ad-FTO^{wt}-oe and ad-pmiRGLO-KCNE1-3'UTR, in 6-well plate. After infection for 6 h, each cell line was re-seeded into a 96-well plate. After 24 h incubation, cells were analyzed with the Dual-Glo Luciferase Assay system (M1000pro, Tecan, Switzerland). Renilla Luciferase (R-luc) was used to normalize firefly luciferase (F-luc) activity to evaluate reporter translation efficiency.

mRNA methylation modification

Total RNA was prepared using TRIzol (Cat# IS10007, Thermo Fisher, USA) as described above. Next, S1 nuclease, alkaline phosphatase and phosphodiesterase I was added to 1 μg RNA diluted in reaction buffer, and the mixture was then incubated at 37°C. After the RNA within the sample was digested, the mixture was extracted with chloroform, and the resulting aqueous layer was collected for analysis with LC-ESI-MS/MS. RNA modification content for each sample was detected by MetWare

(http://www.metware.cn/) based on the AB Sciex QTRAP 6500 LC-MS/MS platform.

m6A sequencing (m6A-seq)

m6A-seq was conducted by Romics (Shanghai, China), according to procedures by the company, Briefly, total RNA was extracted by the Trizol (Thermo Fisher Scientific, 15596018) method. DNase I (Roche Diagnostics, 04716728001) was used to treat Total RNA and remove residual DNA. RNA was fragmented with RNA Fragmentation Buffer (100 mM TrisHCl, 100 mM $ZnCl_2$), and the reaction was terminated by EDTA to recover the fragmented RNA. A portion of the fragmented RNA was taken as Input, and the rest was used for IP. Protein A magnetic beads (Thermo Fisher Scientific, 10002D) and Protein G magnetic beads (Thermo Fisher Scientific, 10004D) were taken and washed twice with IP buffer (150 mM NaCl, 10 mM Tris-HCl [pH 7.5], 0.1% IGEPAL CA-630) was washed twice, and the beads were resuspended in 500ul IP buffer.Add 5ug anti-m6A, rotate and incubate at 4°C for 6h (≥6h) and wash twice with IP buffer. Resuspend beads with 500ul IP Reaction Buffer and incubate at 4°C for 2h. RNA after IP was eluted with the RLT buffer in the RNeasy Mini Kit (QIAGEN, 74106) and purified. Input RNA and IP RNA were library constructed with the SMARTer Stranded Total RNA-Seq Kit-version 2 (Pico Input Mammalian, Takara/Clontech, 634413). Sequencing by Sequencing platform: NovaSeq.

RNA-Seq

RNA-seq was conducted by Romics (Shanghai, China), according to procedures by the company, Briefly, total RNA was extracted by the Trizol (Thermo Fisher Scientific, 15596018) method. Library construction was performed with the NEBNext UltraTM RNA Library Prep Kit (NEB #E7490) and libraries were purified with beads (AMPure XP system). Sequencing platform NovaSeq6000,PE150 system was used for Sequencing.

Oxylipin lipidomics

Oxylipin lipidomics were conducted by MetWare (Wuhan, China), as follows. Atria were removed from mice and then quickly frozen in liquid nitrogen. After processing for lipid extraction, samples were taken for LC-MS/MS analysis. The data acquisition instrumentation system consists primarily of Ultra Performance Liquid Chromatography (UPLC) (ExionLC™ AD) and Tandem Mass

Spectrometry (MS/MS) (QTRAP® 6500+). Eicosanoids contents were detected by MetWare (http://www.metware.cn/) based on the AB Sciex QTRAP 6500 LC-MS/MS platform.

Cell culture, treatments, and adenoviral infection

Adenovirus was used to infect cultured iPSC-aCMs cells for 24 h, and then medium was replaced with fresh medium for another 24 h before cells were harvested. For prostaglandin E2 (PGE2) (Cat# HY-101952, MCE, USA) treatment, 1 μM, 2 μM or 5 μM PGE2 was used to treat cells for 24 h before cells were harvested.

Histopathology

The atria of mice were fixed in 4% PFA for 48 h and paraffin embedded after dehydration, and then 5 mm sections were cut and stained with hematoxylin and eosin staining (H&E) (Cat# G1120, Solarbio, China) or Masson's trichrome stain (Cat# G1340, Solarbio, China).

Immunohistochemistry

Briefly, after deparaffinization and hydration, slides with tissue sections were treated with 0.3% H_2O_2 for 30 min to neutralize endogenous peroxidase, after which the sections were blocked for 2 h at room temperature with 3% blocking serum (Cat# ZLI-9021, zsbio, China) in PBS before incubation with FTO antibody (1:1000, Cat# sc-271713, Santa Cruz Biotechnology, USA) at 4℃ in a moist chamber overnight. Upon incubation with the primary antibody, the specimens were washed three times in phosphate-buffered saline. Immunohistochemistry Universal Kit (Cat# PV-9000, Zsbio, China) was used for secondary antibody incubation and chromography. As a negative control, duplicate sections were immunostained without exposure to primary antibodies.

Immunofluorescence staining

For immunofluorescence staining, the cells were fixed with 4% paraformaldehyde for 15 min, permeabilized with 0.1% Triton X-100 for 10 min, and blocked with 5% bovine serum albumin (BSA) for 30 min. The cells were then incubated with the primary antibody targeting cTni (1:100, Cat# MA1-20112, Invitrogen, USA), atrial-specific marker MLC2a (1:100, Cat# PA5-30789, Invitrogen, USA) and NR2F2 (1:100, Cat# PA5-46850, Invitrogen, USA) overnight at 4℃. After washing three

times with PBS, the cells were incubated with secondary antibody (1:1000, Cat# A-11034 or A-21428, Invitrogen, USA) at room temperature for 1 h. Cell slides were stained with DAPI and then mounted (Cat# P-36931; Invitrogen, USA). Fluorescent images were captured on a laser scanning confocal microscope (TSC SP2; Leica, Germany).

Western blotting

Total proteins from frozen atrial tissues and cells were lysed in lysis buffer (Cat#P0013B, Beytime, China). An SDS-Page gel kit (Cat# P1200, Solarbio, China) and stacking gel (0.125 M Tris-Cl/SDS, pH 6.8; 0.1% SDS; 0.06% ammonium persulfate; 0.1% TEMED and 3.9% acrylamide) was used for protein electrophoresis. Equivalent quantities of protein from the samples were loaded on SDS-Page and ran through the resolving gel for one hour at a constant voltage of 80–100 v and the stacking gel for 2 to 3 h at a constant voltage of 120–200 v. After running, PVDF membranes (Cat# P2938, Sigma-Aldrich, USA) were used for wet transfer at 70 V for 2 h. Next, the PVDF membranes were blocked with 5% skim milk for 1 h and incubated in diluted primary antibodies solutions overnight at 4℃. All of the primary antibodies were diluted in Tris-buffered saline/Tween 20 (TBST) solution containing 5% skim milk. PVDF membrane was incubated with appropriate antibodies: Fto (1:1000, Cat# sc-271713, Santa Cruz Biotechnology, USA), Kcne1 (1:1000, Cat# DF6310, Affinity, China), Kcne2 (1:1000, Cat# PA5-117163, Invitrogen, USA), Kcnq1 (1:1000, Cat# MA5-27676, Invitrogen, USA), Hsp90 (1:2000, Cat# R24635, ZENBIO, China), Wtap (1:2000, Cat# 56501S, CST, USA), Alkb5 (1:2000, Cat# ab195377, Abcam, USA), Mettl3 (1:2000, Cat# 86132S, CST, USA), Mettl14 (1:2000, Cat# 48699S, CST, USA), Gapdh (1:1000, Cat# 92310SF, CST, USA), Ubiquitin (1:2000, Cat# 10201-2-AP, Proteintech, China) at 4℃ overnight, and then incubated with corresponding 800CW-conjugated secondary antibodies (1:10000, Cat# 926-32210, Cat# 926-32211, LI-COR, USA) at room temperature protected from light for 1 h. Membranes were then washed three times before exposure using Odyssey DLx Imaging System (LI-COR, USA). To quantify relative fold change, the images were scanned and band intensity was quantitated using the software Image J.

Quantification and Statistical Analysis

All variables are presented as mean±SD. Normality distribution and variance homogeneity of all statistical values were tested. For variables with normal distribution and homogeneity of variance,

means of two groups were compared by Independent Samples t-tests, whereas means of multiple groups were compared by One way ANOVA or Two way ANOVA (with a Tukey post hoc test, where required). Unpaiblue two-tailed t-tests were used for two-group comparisons of single indicators (young male mice vs. aged male mice, young female mice vs. aged female mice). For multi-group or repeated-measures data, we used two-way ANOVA followed by Tukey's post-hoc test to clarify the main effect of age and potential sex-age interactions. For non-parametric variance models, the Kruskal-Wallis test and Dunn's post hoc multiple comparison tests were used to evaluate statistical significance. Pearson Correlation Analysis was used to measure the similarity or correlation between two data objects. Statistical analyses were performed using Prism 9 (GraphPad Software). *： $p<0.05$; **: $p<0.01$; ***: $p<0.001$; ****: $p<0.0001$;†: p<0.05.

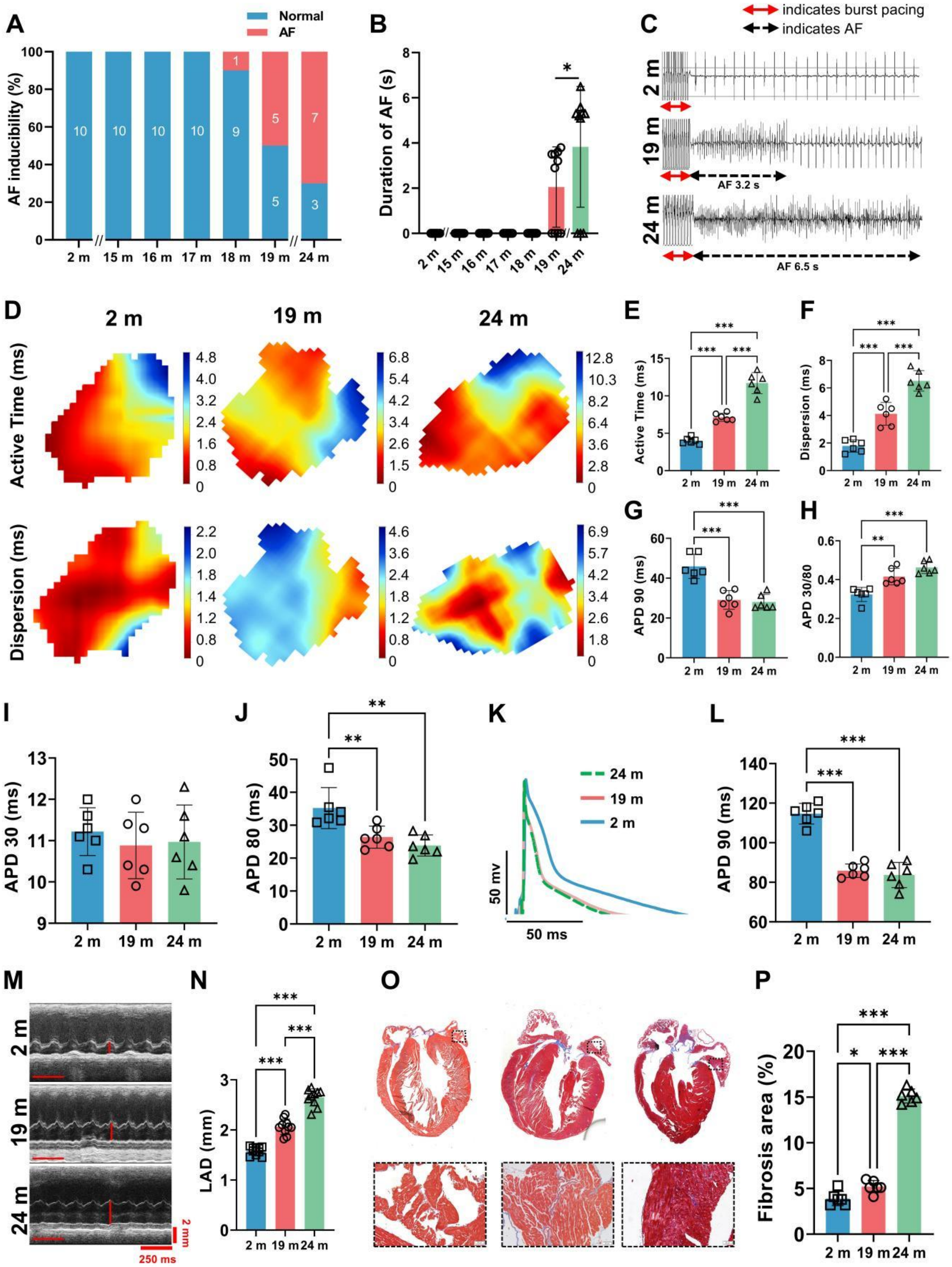

Supplemental figure 1: Aging is associated with increased AF inducibility in mice

A, Inducibility of AF from mice at 2 months, 15-19 months and 24 months of age (n=10/group,). **B,** Total duration of AF from mice at 2 months, 15-19 months and 24 months of age (n=10/group). **C,** Representative intracardiac bipolar electrograms showing induction of AF from mice at 2 months, 19 months and 24 months of age. **D,** Representative optical maps of paced LA from mice at 2 months, 19 months and 24 months of age. **E,** Statistical analysis of active time (n=6/group). **F,** Statistical analysis

of dispersion of conduction (n=6/group). **G,** Statistical analysis of APD90 (n=6/group). **H,** APD30/80 ratio (n=6/group). **I,** Statistical analysis of APD30 (n=6/group). **J,** Statistical analysis of APD80 (n=6/group). **K,** Representative traces of APD in atrial myocytes from mice at 2 months, 19 months and 24 months of age. **L,** Statistical analysis of APD90 (8-10 myocytes/mouse, n=6/group). **M,** Representative images of two-dimensional M-mode for the measurement of LAD at diastole from mice at 2 months, 19 months and 24 months of age, red lines indicate LAD, Time stamp: 250 ms, Vertical bar: 2 mm. **N,** Quantitation of LAD (n=10/group). **O,** Representative images of Masson staining showing fibrotic areas in whole heart and atria from mice at 2 months, 19 months and 24 months of age at low and high magnifications. **P,** Quantitation of percentage of fibrotic area (n=6/group). Data in this figure were analyzed by One-way ANOVA with Turkey's multiple tests. *: $p<0.05$; **: $p<0.01$; ***: $p<0.001$. Male mice were used for the experiment.

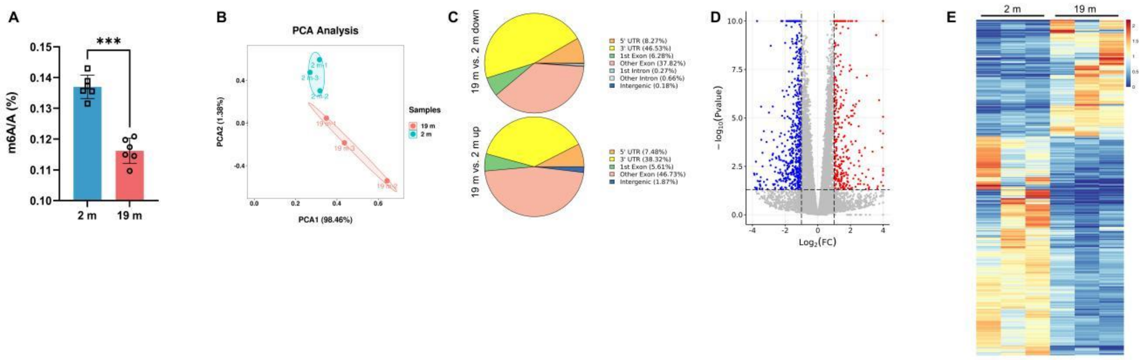

Supplemental figure 2: Aging is associated with m6A down-regulation in the atria

A, Percentage of m6A-methylated RNA in relation to unmodified adenosine as quantified using an antibody-mediated m6A capture assay in the atria from mice at 2 months and at 19 months of age (n=6, Independent Samples t-tests). **B,** PCA analysis of m6A peaks in the atria from mice at 2 months and at 19 months of age. **C,** Distribution of m6A peaks across mRNA regions. **D,** Volcano map of transcriptomics (fold change >1.5 with statistical significance p<0.05). **E,** Heat map of transcriptomics (n=3/group, fold change >2 and false discovery rate (FDR)-adjusted q<0.1). ***: *p*<0.001. Male mice were used for the experiment.

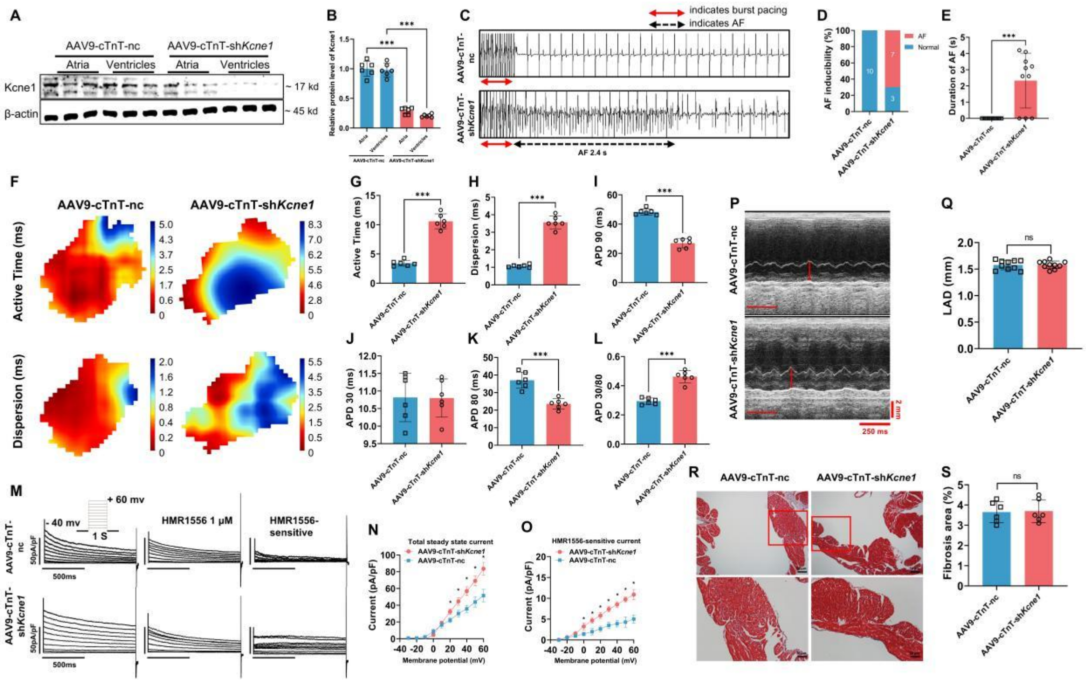

Supplemental figure 3: Cardiomyocyte-specific *Kcne1* knockdown contributes to electrical remodeling

A, Representative immunoblots of Kcne1 protein in the atria or ventricles from mice with or without *Kcne1* knockdown. **B,** Quantification of Kcne1 protein level (n=6/group, Two-way ANOVA with Turkey's multiple tests). **C,** Representative intracardiac bipolar electrograms showing induction of AF from mice with or without *Kcne1* knockdown. **D** and **E,** AF inducibility and total duration of AF (n=10/group, Independent Samples t-tests). **F,** Representative optical maps of paced LA from mice with or without *Kcne1* knockdown. **G,** Statistical analysis of active time (n=6/group, Independent Samples t-tests). **H,** Dispersion of conduction (n=6/group, Independent Samples t-tests). **I,** Statistical analysis of APD90 (n=6/group, Independent Samples t-tests). **J,** Statistical analysis of APD30 (n=6/group, Independent Samples t-tests). **K,** Statistical analysis of APD80 (n=6/group, Independent Samples t-tests). **L,** APD30/80 ratio (n=6/group, Independent Samples t-tests). **M,** Outward current tracings recorded in atrial myocytes (Left), current tracings after exposure to the IKs/IKCNQ1 blocker HMR1556 (Middle), current tracings of HMR1556-sensitive current (Right). **N,** Current-voltage relations of outward current (8-10 myocytes/mouse, n=6/group, Independent Samples t-tests). **O,** Current-voltage relations of HMR1556-sensitive current (8-10 myocytes/mouse, n=6/group, Independent Samples t-tests). **P,** Representative images of two-dimensional M-mode for the measurement of LAD at diastole from mice with or without *Kcne1* knockdown, red lines indicate LAD, Time stamp: 250 ms, Vertical bar: 2 mm. **Q,** Quantitation of LAD (n=10/group, Independent Samples t-tests). **R,** Representative images of Masson staining showing fibrotic area in atria from mice with or without *Kcne1* knockdown at low and high magnifications. **S,** Quantitation of percentage of fibrotic area (n=6/group, Independent Samples t-tests). ***: $p<0.001$. Male mice were used for the experiment.

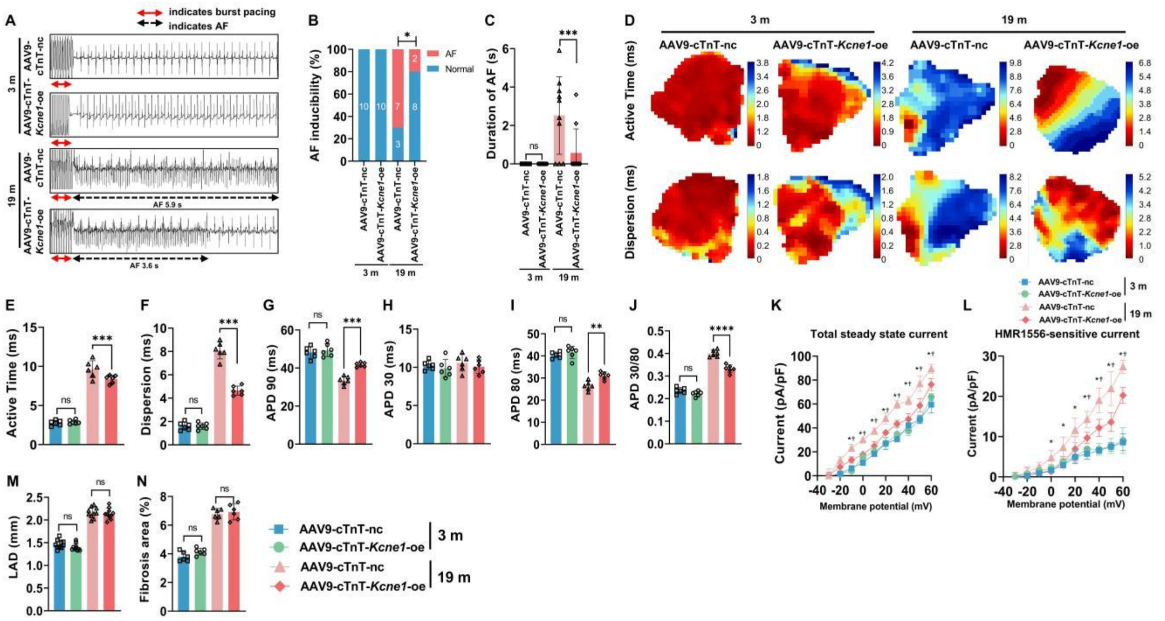

Supplemental figure 4: Aging mice with cardiomyocyte-specific *Kcne1* overexpression reduces aging-induced electrical remodeling

A, Representative intracardiac bipolar electrograms showing induction of AF from mice with or without *Kcne1* overexpression. **B** and **C,** AF inducibility and total duration of AF (n=10/group). **D,** Representative optical maps of paced LA from mice with or without *Kcne1* overexpression. **E,** Statistical analysis of active time (n=6/group). **F,** Dispersion of conduction (n=6/group). **G,** Statistical analysis of APD90 (n=6/group). **H,** Statistical analysis of APD30 (n=6/group). **I,** Statistical analysis of APD80 (n=6/group). **J,** Statistical analysis of APD30/80 ratio (n=6/group). **K,** Current-voltage relations of outward current (8-10 myocytes/mouse, n=6/group). **L,** Current-voltage relations of HMR1556-sensitive current (8-10 myocytes/mouse, n=6/group). **M,** Quantitation of LAD (n=10/group). **N,** Quantitation of percentage of atrial fibrotic area (n=6/group). Data in this figure were analyzed by Two-way ANOVA with Turkey's multiple tests. *: $p<0.05$; **: $p<0.01$; ***: $p<0.001$; ****: $p<0.0001$. (*: 19 m-AAV9-cTnT-*Kcne1*-oe vs 19 m-AAV9-cTnT-nc ($p<0.05$); †: 19 m-AAV9-cTnT-nc vs 3 m-AAV9-cTnT-nc ($p<0.05$) in K and L). Male mice were used for the experiment.

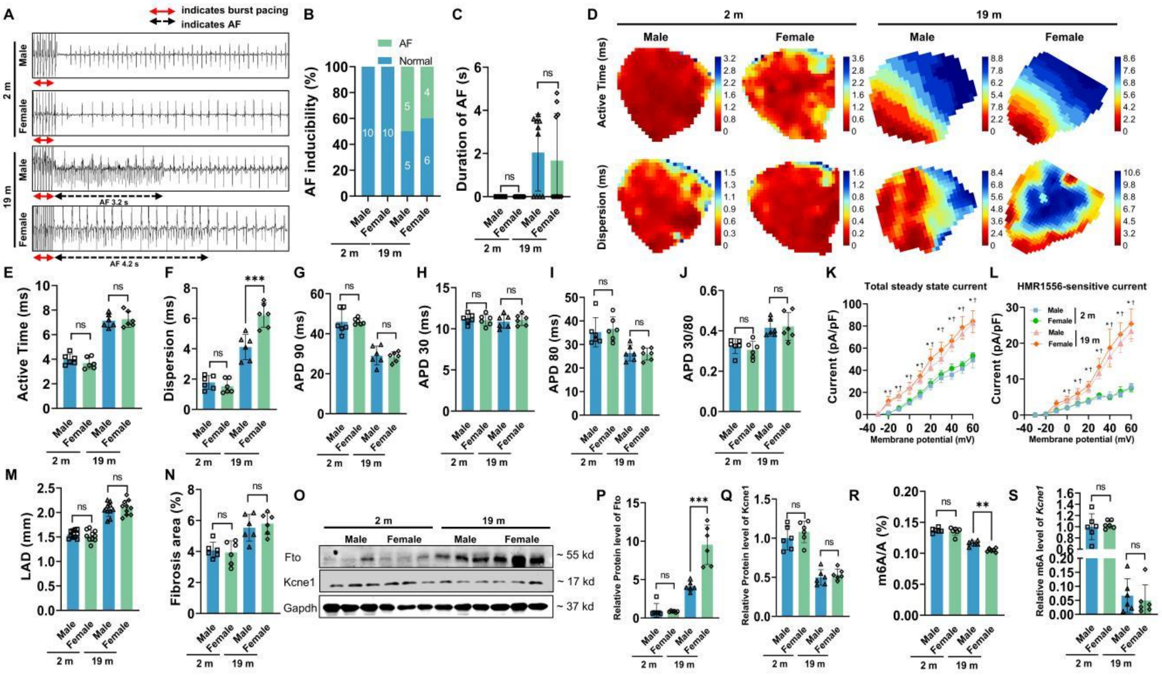

Supplemental figure 5: Validation of aging-induced electrical remodeling and *Kcne1* m6A demethylating in female mice

A, Representative intracardiac bipolar electrograms showing induction of AF from both male and female mice at 2-month-old and 19-month-old. **B** and **C,** AF inducibility and total duration of AF (n=10/group). **D,** Representative optical maps of paced LA from both male and female mice at 2-month-old and 19-month-old. **E,** Statistical analysis of active time (n=6/group). **F,** Dispersion of conduction (n=6/group). **G,** Statistical analysis of APD90 (n=6/group). **H,** Statistical analysis of APD30 (n=6/group). **I,** Statistical analysis of APD80 (n=6/group). **J,** Statistical analysis of APD30/80 ratio (n=6/group). **K,** Current-voltage relations of outward current (8-10 myocytes/mouse, n=6/group). **L,** Current-voltage relations of HMR1556-sensitive current (8-10 myocytes/mouse, n=6/group). **M,** Quantitation of LAD (n=10/group). **N,** Quantitation of percentage of fibrotic area (n=6/group). **O,** Representative immunoblots of Fto and Kcne1 protein in the atria of male and female mice at 2 months and 19 months of age. **P,** Quantification of Fto protein level (n=6/group). **Q,** Quantification of Kcne1 protein level (n=6/group). **R**, Percentage of m6A-methylated RNA in the atria from male and femlae mice at the similar age (n=6/group). **S,** Statistical analysis of m6A levels of *Kcne1* mRNA from male and femlae mice at the similar age (n=6/group). Data in this figure were analyzed by Two-way ANOVA with Turkey's multiple tests. **: $p<0.01$; ***: $p<0.001$. (*: 19 m-Male vs 2 m-Male ($p<0.05$); †: 19 m-Female vs 2 m-Female ($p<0.05$) in K and L). Equal numbers of male and female mice were used for the experiment.

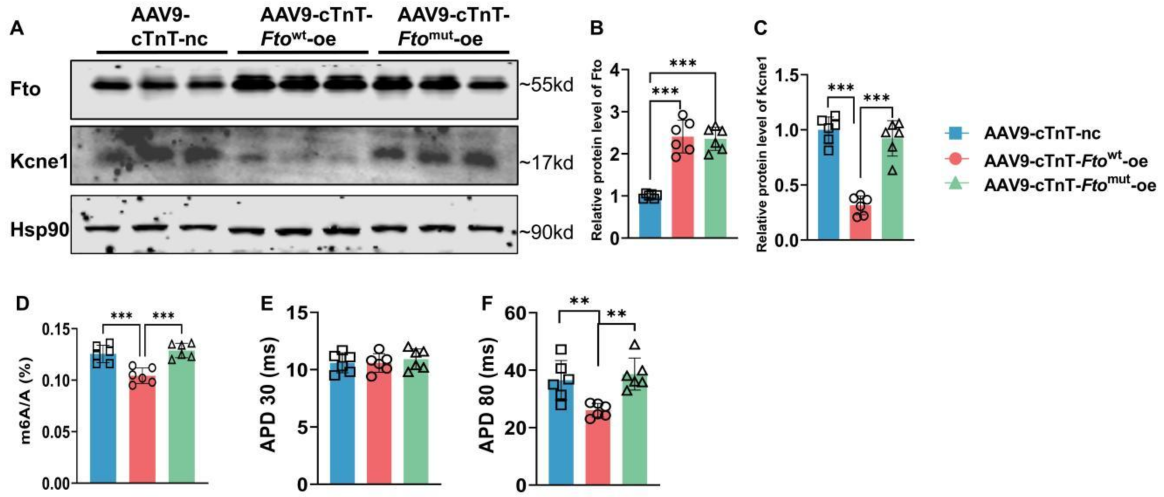

Supplemental figure 6: Levels of Kcne1 m6A and protein are influenced by *Fto* overexpression in mouse atrial tissues

A, Representative immunoblots of Fto and Kcne1 protein in the atria of mice injected with AAV9-cTnT-*Fto*^{wt}-oe, AAV9-cTnT-*Fto*^{mut}-oe and AAV9-cTnT-nc. **B,** Quantification of Fto protein level (n=6/group). **C,** Quantification of Kcne1 protein level (n=6/group). **D,** Percentage of m6A-methylated RNA in the atria from mice injected with AAV9-cTnT-*Fto*^{wt}-oe, AAV9-cTnT-*Fto*^{mut}-oe and AAV9-cTnT-nc (n=6/group). **E,** Statistical analysis of APD30 (n=6/group). **F,** Statistical analysis of APD80 (n=6/group). Data in this figure were analyzed by One-way ANOVA with Turkey's multiple tests. **: p<0.01; ***: p<0.001. Male mice were used for the experiment.

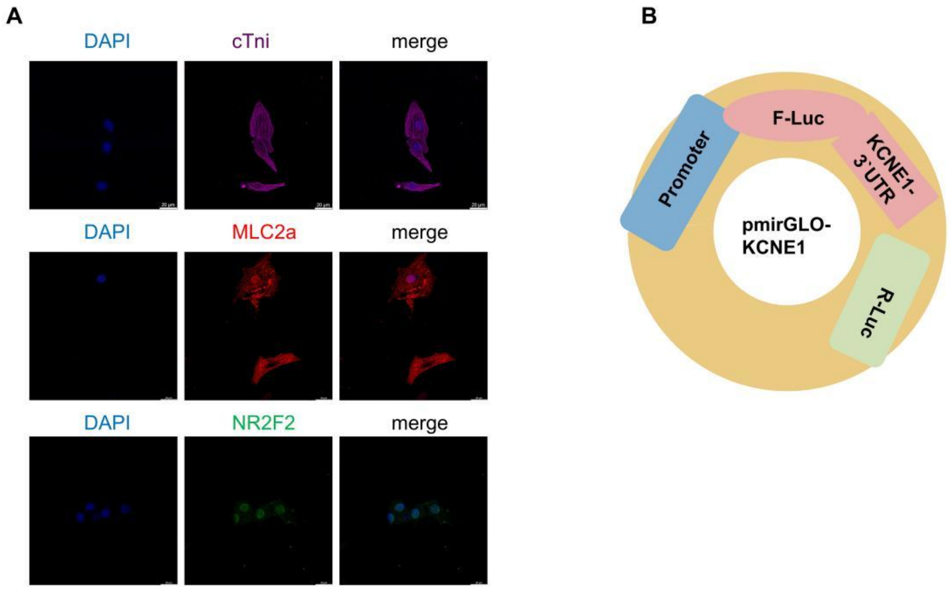

Supplemental figure 7: Confirmation of cardiomyocyte differentiation and summary illustration of a KCNE1 luciferase reporter plasmid

A, Representative immunofluorescence staining of cardiac-specific marker cTni and atrial-specific marker (MLC2a and NR2F2) in iPSC-aCMs. **B,** iPSC-aCMs with overexpression of *FTO*^{wt}, *FTO*^{mut} or blank vector were infected with pmirGLO-*KCNE1*-3'UTR reporter vector for 24 h.

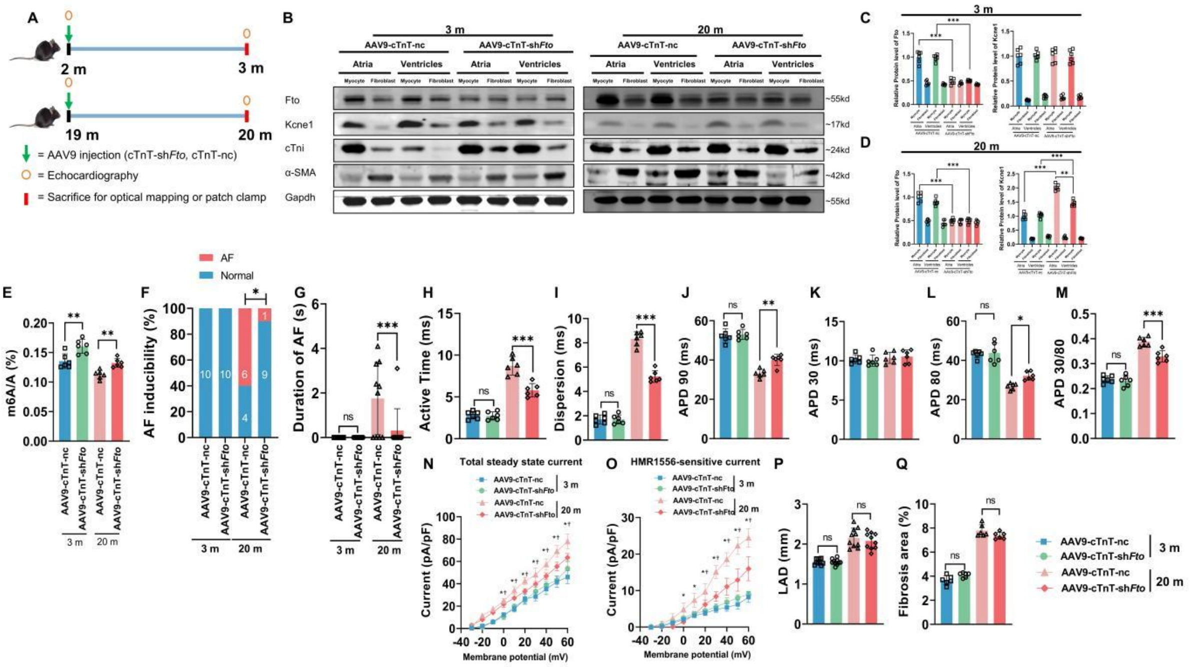

Supplemental figure 8: Genetic deletion of Fto alleviates aging-induced AF

A, The experimental design of the study. B, Representative immunoblots of Fto and Kcne1 protein in the cardiomyocytes or fibroblasts of atria or ventricles of adult mice and aged mice injected with AAV9-cTnT-shFto and AAV9-cTnT-nc (n=6/group). C, Quantification of Fto and Kcne1 protein level in adult mice (n=6/group). D, Quantification of Fto and Kcne1 protein level in aged mice (n=6/group). E, Statistical analysis of total m6A levels in the atria from adult mice and aged mice injected with AAV9-cTnT-shFto and AAV9-cTnT-nc (n=6/group). F and G, AF inducibility and total duration of AF (n=10/group). H, Statistical analysis of active time (n=6/group). I, Dispersion of conduction (n=6/group). J, Statistical analysis of APD90 (n=6/group). K, Statistical analysis of APD30 (n=6/group). L, Statistical analysis of APD80 (n=6/group). M, APD30/80 ratio (n=6/group). N, Current-voltage relations of outward current (8-10 myocytes/mouse, n=6/group). O, Current-voltage relations of HMR1556-sensitive current (8-10 myocytes/mouse, n=6/group). P, Quantitation of LAD (n=10/group). Q, Quantitation of percentage of atrial fibrotic area (n=6/group). Data in this figure were analyzed by Two-way ANOVA with Turkey's multiple tests. *: p<0.05; **: p<0.01; ***: p<0.001. (*: 20 m-AAV9-cTnT-shFto vs 20 m-AAV9-cTnT-nc (p<0.05); †: 20 m-AAV9-cTnT-nc vs 3 m-AAV9-cTnT-nc (p<0.05) in N and O). Male mice were used for the experiment.

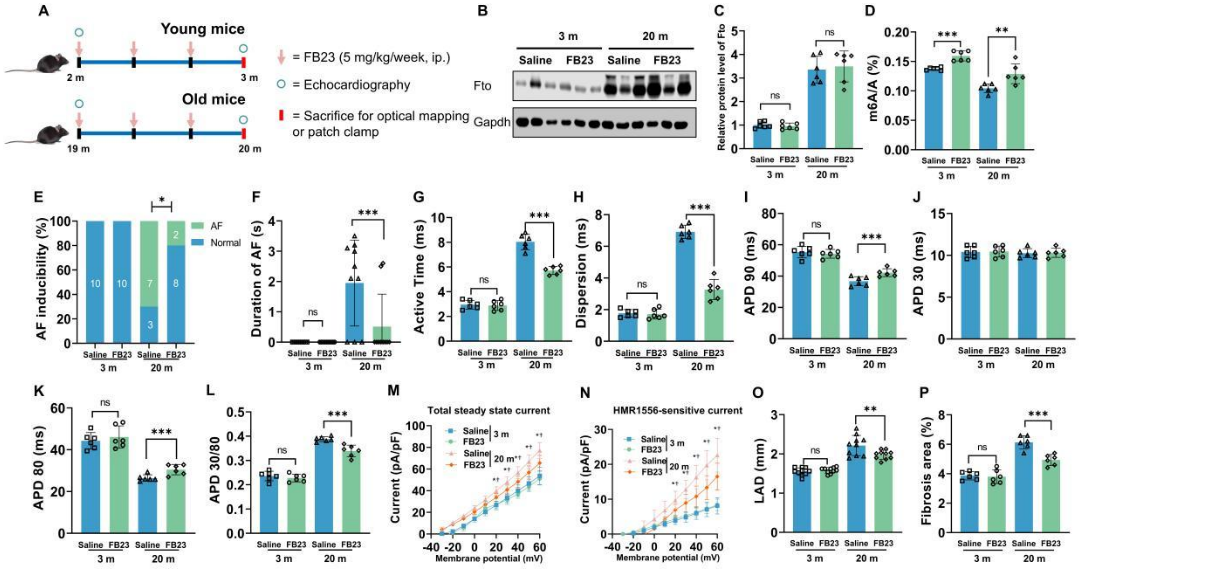

Supplemental figure 9: Pharmacological inhibition of Fto demethylase activity alleviates aging-induced AF in mice

A, The experimental design of the study. **B,** Representative immunoblots of Fto in the atria of adult mice or aged mice subjected to saline and FB23 (n=6/group). **C,** Quantification of Fto protein level (n=6/group). **D,** Statistical analysis of total m6A levels in the atria from adult mice or aged mice subjected to saline and FB23 (n=6/group). **E** and **F,** AF inducibility and total duration of AF (n=10/group). **G,** Statistical analysis of active time (n=6/group). **H,** Dispersion of conduction (n=6/group). **I,** Statistical analysis of APD90 (n=6/group). **J,** Statistical analysis of APD30 (n=6/group). **K,** Statistical analysis of APD80 (n=6/group). **L,** APD30/80 ratio (n=6/group). **M,** Current-voltage relations of outward current (8-10 myocytes/mouse, n=6/group). **N,** Current-voltage relations of HMR1556-sensitive current (8-10 myocytes/mouse, n=6/group). **O,** Quantitation of LAD (n=10/groups). **P,** Quantitation of percentage of atrial fibrotic area (n=6/group). Data in this figure were analyzed by Two-way ANOVA with Turkey's multiple tests. *: p<0.05; **: p<0.01; ***: p<0.001. (*: 20 m-FB23 vs 20 m-Saline (p<0.05); †: 20 m-Saline vs 3 m-Saline (p<0.05) in M and N). Male mice were used for the experiment.

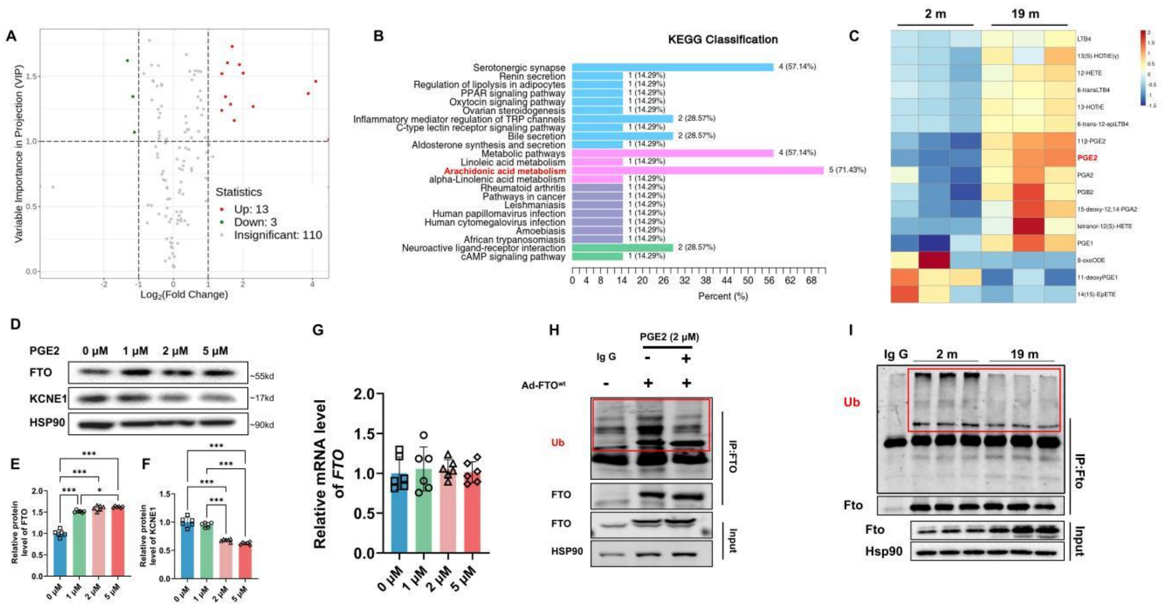

Supplemental figure 10: Persistence of PGE2 in aging atria represses FTO degradation by inhibiting the ubiquitination of FTO

A, Volcano plot of oxylipins of atria from mice at 2 months and 19 months of age. Significantly up-regulated (red) and down-regulated (green) enrichment is highlighted (n=3/group, fold change >1.5 with statistical significance p<0.05). **B,** KEGG classification of 126 oxylipins. **C,** Heatmap of 16 oxylipins with significantly statistical difference (n=3/group, fold change >2 and false discovery rate (FDR)-adjusted q<0.1). **D,** Representative immunoblots of FTO and KCNE1 protein of iPSC-aCMs treated with PGE2. **E,** Quantification of FTO protein level (n=6/group, One-way ANOVA with Turkey's multiple tests). **F,** Quantification of KCNE1 protein level (n=6/group, One-way ANOVA with Turkey's multiple tests). **G,** FTO mRNA level of iPSC-aCMs treated with PGE2 (n=6/group, One-way ANOVA with Turkey's multiple tests). **H,** Representative immunoblotting of ubiquitinated FTO from the IP assay of iPSC-aCMs treated with PGE2 (n=3/group). **I,** Representative immunoblotting of ubiquitinated FTO from the IP assay of atria from mice at 2 months and 19 months of age (n=3/group). *: *p*<0.05; ***: *p*<0.001. Male mice were used for the experiment.

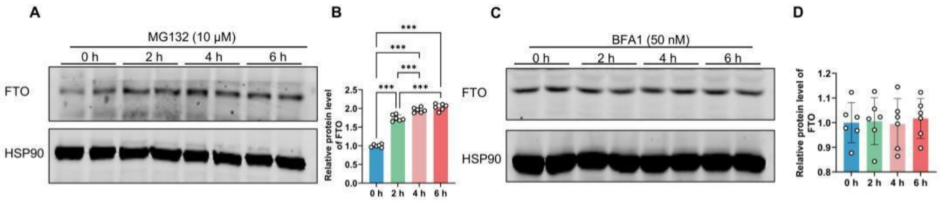

Supplemental figure 11: FTO degradation via the ubiquitin proteasome pathway

A, Representative immunoblots of FTO protein of iPSC-aCMs treated with MG132 for the indicated times. **B,** Quantification of FTO protein level (n=6/group). **C,** Representative immunoblots of FTO protein of iPSC-aCMs treated with BFA1 for the indicated times. **D,** Quantification of FTO protein level (n=6/group). Data in this figure were analyzed by One-way ANOVA with Turkey's multiple tests. ***: p<0.001.

Supplemental table 1

	2 m	19 m	24 m
BW (g)	25.3±2.3	36.6±3.3*	35.2±2.1*†
HW/TL (mg/mm)	6.44±0.23	8.62±0.50*	8.79±0.54*
HW/BW (mg/g)	4.32±0.19	4.25±0.11	4.34±0.14
MBP (mmHg)	103.2±6.3	116.2±5.9	114.9±6.6
HR (bpm)	526.3±11.5	513.6±19.5	532.6±30.5
LV mass (mg)	96.8±6.4	120.5±10.5*	118.9±9.6*
LVEF (%)	64.5±4.2	68.9±3.2	66.8±4.1
LVFS (%)	34.5±5.1	37.5±3.2	36.4±3.5
LVPWd (mm)	0.86±0.23	0.87±0.31	0.85±0.34
LVPWs (mm)	1.63±0.32	1.56±0.22	1.54±0.34
LVAWd (mm)	0.88±0.19	1.57±0.41*	1.62±0.33*
LVAWs (mm)	1.19±0.15	1.94±0.24*	1.89±0.36*
LVDs (mm)	2.14±0.35	2.49±0.18*	2.52±0.21*
LVDd (mm)	3.41±0.24	3.75±0.14*	3.82±0.24*

BW: Body Weight; HW/TL: Heart Weight /Tibia Length; HW/BW: Heart Weight/Body Weight; MBP: Mean Blood Pressure; HR: Heart Rate; LV mass: Left Ventricular mass; LVEF: Left Ventricle Ejection Fraction; LVFS: Left Ventricle Fractional Shortening; LVPWd: Thickness of Left Ventricular Posterior Wall at end-diastolic phase; LVPWs: Thickness of Left Posterior Wall at end-systolic phase; LVAWd: Thickness of Left Ventricular Anterior Wall at end-diastolic phase; LVAWs: Thickness of Left Ventricular Anterior Wall at end-systolic phase; LVDs: Left Ventricular Internal Diameter at end-systolic phase; LVDd: Left Ventricular Internal Diameter at end-diastolic phase. (n=10/group, One-way ANOVA with Turkey's multiple tests). *: vs 2 m ($P < 0.05$); †: vs 19 m ($P < 0.05$).

Supplemental table 2

	AAV9-cTnT-nc	AAV9-cTnT-sh*Kcne1*
BW (g)	26.5±2.1	26.1±2.1
HW/TL (mg/mm)	6.84±0.34	6.79±0.21
HW/BW (mg/g)	4.21±0.17	4.17±0.18
MBP (mmHg)	119.5±3.6	121.6±5.6
HR (bpm)	521.3±23.6	524.6±25.6
LV mass (mg)	95.6±5.6	96.8±7.4
LVEF (%)	64.6±3.3	63.8±1.7
LVFS (%)	35.6±5.4	36.9±4.3
LVPWd (mm)	0.87±0.13	0.85±0.14
LVPWs (mm)	1.50±0.21	1.48±0.16
LVAWd (mm)	0.81±0.21	0.82±0.11
LVAWs (mm)	1.29±0.17	1.31±0.19
LVDs (mm)	1.86±0.24	1.91±0.37
LVDd (mm)	3.27±0.16	3.25±0.31

BW: Body Weight; HW/TL: Heart Weight /Tibia Length; HW/BW: Heart Weight/Body Weight; MBP: Mean Blood Pressure; HR: Heart Rate; LV mass: Left Ventricular mass; LVEF: Left Ventricle Ejection Fraction; LVFS: Left Ventricle Fractional Shortening; LVPWd: Thickness of Left Ventricular Posterior Wall at end-diastolic phase; LVPWs: Thickness of Left Posterior Wall at end-systolic phase; LVAWd: Thickness of Left Ventricular Anterior Wall at end-diastolic phase; LVAWs: Thickness of Left Ventricular Anterior Wall at end-systolic phase; LVDs: Left Ventricular Internal Diameter at end-systolic phase; LVDd: Left Ventricular Internal Diameter at end-diastolic phase. (n=10/group, Independent Samples t-tests).

Supplemental table 3

	3 m		19 m	
	AAV9-cTnT-nc	AAV9-cTnT-*Kcne1*-oe	AAV9-cTnT-nc	AAV9-cTnT-*Kcne1*-oe
BW (g)	26.6±1.9	25.9±2.4	34.2±3.4*	35.7±2.7*
HW/TL (mg/mm)	6.57±0.45	6.62±0.34	8.62±0.24*	8.71±0.26*
HW/BW (mg/g)	4.27±0.20	4.24±0.22	4.23±0.21	4.21±0.17
MBP (mmHg)	110.5±17.5	115.5±18.3	118.8±23.6	120.8±22.8
HR (bpm)	534.1±20.7	529.6±19.5	534.4±22.9	523.6±24.5
LV mass (mg)	93.2±4.4	95.6±5.1	120.9±6.1*	118.7±5.2*
LVEF (%)	64.7±2.7	65.9±3.7	65.1±4.4	66.8±5.4
LVFS (%)	33.6±2.8	35.1±4.2	34.2±5.1	35.2±5.6
LVPWd (mm)	0.89±0.14	0.86±0.21	0.84±0.32	0.85±0.29
LVPWs (mm)	1.48±0.21	1.53±0.30	1.55±0.27	1.52±0.34
LVAWd (mm)	0.85±0.32	0.84±0.28	1.58±0.34*	1.56±0.28*
LVAWs (mm)	1.42±0.37	1.37±0.23	1.79±0.22*	1.81±0.34*
LVDs (mm)	2.17±0.21	2.24±0.24	2.68±0.17*	2.59±0.27*
LVDd (mm)	3.31±0.17	3.25±0.24	3.67±0.23*	3.64±0.34*

BW: Body Weight; HW/TL: Heart Weight /Tibia Length; HW/BW: Heart Weight/Body Weight; MBP: Mean Blood Pressure; HR: Heart Rate; LV mass: Left Ventricular mass; LVEF: Left Ventricle Ejection Fraction; LVFS: Left Ventricle Fractional Shortening; LVPWd: Thickness of Left Ventricular Posterior Wall at end-diastolic phase; LVPWs: Thickness of Left Posterior Wall at end-systolic phase; LVAWd: Thickness of Left Ventricular Anterior Wall at end-diastolic phase; LVAWs: Thickness of Left Ventricular Anterior Wall at end-systolic phase; LVDs: Left Ventricular Internal Diameter at end-systolic phase; LVDd: Left Ventricular Internal Diameter at end-diastolic phase. (n=10/group, Two-way ANOVA with Turkey's multiple tests). *: vs 3 m-AAV9-cTnT-nc ($P < 0.05$).

Supplemental Table 4

Variables	Non-AF (n=12)	AF (n=12)	p value
Age (years)	64.62±10.52	65.66±8.75	0.774
Male (n, %)	8 (66.7%)	7 (58.3%)	0.726
Smoking (n, %)	8 (66.7%)	7 (58.3%)	0.726
**Medical history**			
Hyperlipidemia (n, %)	5 (41.6%)	6 (50%)	0.614
Hypertension (n, %)	8 (66.7%)	7 (58.3%)	0.726
**Medication**			
Beta blockers (n, %)	10 (83.3%)	11 (91.6%)	1.000
ACEI (n, %)	8 (66.7%)	8 (66.7%)	1.000
**Lab tests of plasma**			
Cre (μmol/L)	72.47±43.62	74.03±23.18	0.915
UA (μmol/L)	363.17±90.52	361.59±80.37	0.959
FBG (mmol/L)	5.54±1.02	5.58±1.35	0.935
FIB (g/L)	5.75±1.18	5.62±1.08	0.786
TC (mmol/L)	4.63±1.12	4.51±1.04	0.982
HDL (mmol/L)	2.09±0.14	2.11±0.16	0.526
HCY (μmol/L)	13.42±4.21	13.50±3.38	0.370
TSH (mU/L)	2.21±1.03	2.19±1.01	0.473
**Echocardiography**			
LVEF (%)	63.23±4.08	62.63±5.21	0.441
LAVI (mL/m^2)	33.24±1.98	37.59±2.01	0.035

ACEI: angiotensin-converting enzyme inhibitors; Cre: creatinine; UA: uric acid; FBG: Fasting Blood Glucose; FIB: fibrinogen; TC: total cholesterol; HDL: high-density lipoprotein; HCY: homocysteine; TSH: thyroid stimulating hormone; LVEF: left ventricular ejection fraction; LAVI: Left Atrial Volume Index.

Supplemental table 5

	2 m		19 m	
	Male	Female	Male	Female
BW (g)	26.8±1.01	22.2±1.31*	33.4±1.1*	30.6±0.96†#
HW/TL (mg/mm)	6.52±0.27	6.62±0.34	8.82±0.58*	8.53±0.42†
HW/BW (mg/g)	4.21±0.11	4.22±0.31	4.24±0.34	4.43±0.21
MBP (mmHg)	108.8±13.1	105.6±14.8	107.9±15.2	109.6±10.3
HR (bpm)	538.4±16.7	529.7±22.6	536.4±18.5	528.9±21.4
LV mass (mg)	94.6±7.5	96.5±6.4	109.8±8.6*	108.7±10.8†
LVEF (%)	64.9±3.8	65.7±8.1	64.2±7.2	65.5±4.9
LVFS (%)	34.2±5.4	33.6±3.8	35.7±4.3	34.5±5.6
LVPWd (mm)	0.91±0.28	0.83±0.16	0.88±0.35	0.87±0.26
LVPWs (mm)	1.54±0.54	1.43±0.43	1.54±0.42	1.53±0.17
LVAWd (mm)	0.95±0.34	0.92±0.17	1.28±0.18*	1.34±0.24†
LVAWs (mm)	1.29±0.12	1.31±0.18	1.69±0.22*	1.61±0.31†
LVDs (mm)	2.14±0.39	2.35±0.44	2.61±0.18*	2.59±0.11†
LVDd (mm)	3.35±0.42	3.46±0.35	3.92±0.31*	3.84±0.27†

BW: Body Weight; HW/TL: Heart Weight /Tibia Length; HW/BW: Heart Weight/Body Weight; MBP: Mean Blood Pressure; HR: Heart Rate; LV mass: Left Ventricular mass; LVEF: Left Ventricle Ejection Fraction; LVFS: Left Ventricle Fractional Shortening; LVPWd: Thickness of Left Ventricular Posterior Wall at end-diastolic phase; LVPWs: Thickness of Left Posterior Wall at end-systolic phase; LVAWd: Thickness of Left Ventricular Anterior Wall at end-diastolic phase; LVAWs: Thickness of Left Ventricular Anterior Wall at end-systolic phase; LVDs: Left Ventricular Internal Diameter at end-systolic phase; LVDd: Left Ventricular Internal Diameter at end-diastolic phase. (n=10/group, Two-way ANOVA with Turkey's multiple tests). *: vs 2 m-Male ($P < 0.05$); †: vs 2 m-Female ($P < 0.05$); #: vs 19 m-Male ($P < 0.05$).

Supplemental table 6

	2 m		19 m	
	Cre$^-$;*Fto*$^{fl/fl}$	*Cre*$^+$;*Fto*$^{fl/fl}$	*Cre*$^-$;*Fto*$^{fl/fl}$	*Cre*$^+$;*Fto*$^{fl/fl}$
BW (g)	24.3±6.2	25.1±4.2	33.4±2.7*	33.9±4.6*
HW/TL (mg/mm)	6.53±0.31	6.47±0.29	8.62±0.43*	8.05±0.24*†
HW/BW (mg/g)	4.21±0.18	4.23±0.20	4.24±0.22	4.18±0.23
MBP (mmHg)	110.3±4.5	108.6±13.5	112.6±18.1	108.5±18.7
HR (bpm)	513.6±13.5	520.3±14.6	506.2±16.3	520.6±20.5
LV mass (mg)	96.5±4.4	96.1±3.2	116.4±3.0*	108.8±3.6*†
LVEF (%)	66.5±3.8	65.7±4.3	66.4±3.9	67.2±4.3
LVFS (%)	35.2±4.1	34.9±2.7	35.4±2.1	36.7±5.1
LVPWd (mm)	0.86±0.18	0.87±0.17	0.90±0.15	0.90±0.19
LVPWs (mm)	1.54±0.15	1.53±0.26	1.58±0.16	1.56±0.24
LVAWd (mm)	0.97±0.18	0.93±0.24	1.52±0.15*	1.42±0.23*
LVAWs (mm)	1.36±0.29	1.31±0.14	1.76±0.11*	1.53±0.17*†
LVDs (mm)	2.27±0.12	2.16±0.15	2.78±0.14*	2.73±0.11*
LVDd (mm)	3.21±0.10	3.30±0.17	3.82±0.19*	3.76±0.18*

BW: Body Weight; HW/TL: Heart Weight /Tibia Length; HW/BW: Heart Weight/Body Weight; MBP: Mean Blood Pressure; HR: Heart Rate; LV mass: Left Ventricular mass; LVEF: Left Ventricle Ejection Fraction; LVFS: Left Ventricle Fractional Shortening; LVPWd: Thickness of Left Ventricular Posterior Wall at end-diastolic phase; LVPWs: Thickness of Left Posterior Wall at end-systolic phase; LVAWd: Thickness of Left Ventricular Anterior Wall at end-diastolic phase; LVAWs: Thickness of Left Ventricular Anterior Wall at end-systolic phase; LVDs: Left Ventricular Internal Diameter at end-systolic phase; LVDd: Left Ventricular Internal Diameter at end-diastolic phase. (n=10/group, Two-way ANOVA with Turkey's multiple tests). *: vs 2 m *Cre*$^-$;*Fto*$^{fl/fl}$ ($P < 0.05$); †: vs 19 m *Cre*$^-$;*Fto*$^{fl/fl}$ ($P < 0.05$).

Supplemental table 7

	AAV9-cTnT-nc	AAV9-cTnT-*Fto*^{wt}-oe	AAV9-cTnT-*Fto*^{mut}-oe
BW (g)	24.9±3.7	25.2±4.3	25.8±4.4
HW/TL (mg/mm)	6.59±0.24	6.75±0.21	6.63±0.31
HW/BW (mg/g)	4.32±0.24	4.41±0.09	4.24±0.18
MBP (mmHg)	112.6±20.3	108.6±17.5	110.5±16.9
HR (bpm)	523.6±19.8	519.6±24.5	526.3±19.7
LV mass (mg)	92.6±4.1	95.8±6.4	94.6±3.7
LVEF (%)	65.3±3.4	66.1±7.1	65.9±4.4
LVFS (%)	34.6±4.3	34.8±6.4	35.6±3.2
LVPWd (mm)	0.84±0.14	0.82±0.15	0.86±0.42
LVPWs (mm)	1.62±0.32	1.66±0.48	1.63±0.45
LVAWd (mm)	0.86±0.42	0.88±0.33	0.84±0.47
LVAWs (mm)	1.26±0.30	1.34±0.18	1.26±0.41
LVDs (mm)	2.15±0.18	2.24±0.35	2.19±0.21
LVDd (mm)	3.42±0.21	3.58±0.33	3.48±0.28

BW: Body Weight; HW/TL: Heart Weight /Tibia Length; HW/BW: Heart Weight/Body Weight; MBP: Mean Blood Pressure; HR: Heart Rate; LV mass: Left Ventricular mass; LVEF: Left Ventricle Ejection Fraction; LVFS: Left Ventricle Fractional Shortening; LVPWd: Thickness of Left Ventricular Posterior Wall at end-diastolic phase; LVPWs: Thickness of Left Posterior Wall at end-systolic phase; LVAWd: Thickness of Left Ventricular Anterior Wall at end-diastolic phase; LVAWs: Thickness of Left Ventricular Anterior Wall at end-systolic phase; LVDs: Left Ventricular Internal Diameter at end-systolic phase; LVDd: Left Ventricular Internal Diameter at end-diastolic phase. (n=10/group, One-way ANOVA with Turkey's multiple tests).

Supplemental table 8

	AAV9-cTnT-nc1		AAV9-cTnT-*Fto*-oe	
	AAV9-cTnT-nc2	AAV9-cTnT-*Kcne1*-oe	AAV9-cTnT-nc2	AAV9-cTnT-*Kcne1*-oe
BW (g)	26.8±3.6	25.9±2.7	27.5±2.4	25.9±4.1
HW/TL (mg/mm)	6.53±0.24	6.64±0.28	6.81±0.28	6.79±0.27
HW/BW (mg/g)	4.32±0.31	4.25±0.24	4.43±0.34	4.41±0.32
MBP (mmHg)	124.5±16.5	118.5±16.4	116.3±9.8	123.1±7.6
HR (bpm)	531.6±26.1	521.6±19.8	518.4±20.3	526.5±28.4
LV mass (mg)	96.5±4.1	96.7±7.2	101.5±8.8	106.9±9.2
LVEF (%)	66.9±2.9	65.4±3.7	67.4±4.8	66.4±3.1
LVFS (%)	35.6±6.4	34.2±3.8	36.4±3.5	35.3±2.1
LVPWd (mm)	0.88±0.24	0.85±0.16	0.86±0.32	0.88±0.26
LVPWs (mm)	1.55±0.24	1.54±0.21	1.54±0.44	1.53±0.31
LVAWd (mm)	0.87±0.424	0.86±0.45	0.89±0.38	0.91±0.41
LVAWs (mm)	1.21±0.31	1.19±0.47	1.23±0.34	1.21±0.47
LVDs (mm)	2.09±0.33	2.13±0.47	2.29±0.32	2.21±0.45
LVDd (mm)	3.24±0.33	3.27±0.45	3.45±0.29	3.52±0.42

BW: Body Weight; HW/TL: Heart Weight /Tibia Length; HW/BW: Heart Weight/Body Weight; MBP: Mean Blood Pressure; HR: Heart Rate; LV mass: Left Ventricular mass; LVEF: Left Ventricle Ejection Fraction; LVFS: Left Ventricle Fractional Shortening; LVPWd: Thickness of Left Ventricular Posterior Wall at end-diastolic phase; LVPWs: Thickness of Left Posterior Wall at end-systolic phase; LVAWd: Thickness of Left Ventricular Anterior Wall at end-diastolic phase; LVAWs: Thickness of Left Ventricular Anterior Wall at end-systolic phase; LVDs: Left Ventricular Internal Diameter at end-systolic phase; LVDd: Left Ventricular Internal Diameter at end-diastolic phase. (n=10/group, Two-way ANOVA with Turkey's multiple tests).

Supplemental table 9

	3 m		20 m	
	AAV9-cTnT-nc	AAV9-cTnT-sh*Fto*	AAV9-cTnT-nc	AAV9-cTnT-sh*Fto*
BW (g)	26.2±3.8	27.1±3.3	35.2±2.4*	34.9±2.7*
HW/TL (mg/mm)	6.65±0.27	6.59±0.32	8.03±0.28*	7.95±0.15*
HW/BW (mg/g)	4.10±0.19	4.22±0.18	4.26±0.18*	4.24±0.11*
MBP (mmHg)	112.3±22.5	108.4±16.3	114.6±15.2	116.8±218.7
HR (bpm)	503.6±18.7	512.3±24.1	522.8±22.4	517.9±23.9
LV mass (mg)	93.5±4.1	96.4±3.7	118.6±4.2*	116.9±3.7*
LVEF (%)	66.0±4.2	65.1±3.8	64.1±4.5	64.3±4.7
LVFS (%)	36.3±4.7	34.5±4.3	35.4±6.6	34.9±3.3
LVPWd (mm)	0.85±0.10	0.83±0.15	0.83±0.12	0.84±0.08
LVPWs (mm)	1.47±0.15	1.49±0.20	1.52±0.21	1.57±0.11
LVAWd (mm)	0.86±0.15	0.85±0.12	1.47±0.21*	1.46±0.25*
LVAWs (mm)	1.44±0.13	1.38±0.15	1.88±0.19*	1.83±0.22*
LVDs (mm)	2.09±0.25	2.15±0.27	2.68±0.11*	2.62±0.17*
LVDd (mm)	3.27±0.24	3.19±0.18	3.79±0.25*	3.75±0.21*

BW: Body Weight; HW/TL: Heart Weight /Tibia Length; HW/BW: Heart Weight/Body Weight; MBP: Mean Blood Pressure; HR: Heart Rate; LV mass: Left Ventricular mass; LVEF: Left Ventricle Ejection Fraction; LVFS: Left Ventricle Fractional Shortening; LVPWd: Thickness of Left Ventricular Posterior Wall at end-diastolic phase; LVPWs: Thickness of Left Posterior Wall at end-systolic phase; LVAWd: Thickness of Left Ventricular Anterior Wall at end-diastolic phase; LVAWs: Thickness of Left Ventricular Anterior Wall at end-systolic phase; LVDs: Left Ventricular Internal Diameter at end-systolic phase; LVDd: Left Ventricular Internal Diameter at end-diastolic phase. (n=10/group, Two-way ANOVA with Turkey's multiple tests). *: vs 3 m-AAV9-cTnT-nc($P < 0.05$).

Supplemental table 10

	3 m		20 m	
	Saline	FB 23	Saline	FB 23
BW (g)	25.2±2.3	25.9±1.7	36.5±4.3*	28.9±1.2*†
HW/TL (mg/mm)	6.62±0.25	6.57±0.33	8.81±0.28*	7.94±0.14*†
HW/BW (mg/g)	4.32±0.05	4.18±0.22	4.27±0.10	4.23±0.08
MBP (mmHg)	115.3±20.3	111.6±17.4	108.4±16.4	117.6±20.4
HR (bpm)	520.2±17.5	518.3±20.4	523.6±28.4	518.9±22.9
LV mass (mg)	96.5±4.1	95.7±3.4	127.4±3.6*	118.3±3.2*†
LVEF (%)	68.5±3.1	66.7±3.4	68.4±2.4	67.4±4.1
LVFS (%)	36.4±2.4	34.7±2.1	36.1±4.4	35.7±2.5
LVPWd (mm)	0.84±0.24	0.81±0.33	0.82±0.26	0.85±0.32
LVPWs (mm)	1.55±0.31	1.63±0.47	1.66±0.34	1.61±0.32
LVAWd (mm)	0.95±0.22	0.98±0.47	1.63±0.15*	1.29±0.12*†
LVAWs (mm)	1.32±0.18	1.37±0.14	1.94±0.13*	1.52±0.16*†
LVDs (mm)	2.16±0.25	2.28±0.14	2.94±0.15*	2.63±0.17*†
LVDd (mm)	3.37±0.34	3.21±0.12	3.93±0.25*	3.68±0.12*†

BW: Body Weight; HW/TL: Heart Weight /Tibia Length; HW/BW: Heart Weight/Body Weight; MBP: Mean Blood Pressure; HR: Heart Rate; LV mass: Left Ventricular mass; LVEF: Left Ventricle Ejection Fraction; LVFS: Left Ventricle Fractional Shortening; LVPWd: Thickness of Left Ventricular Posterior Wall at end-diastolic phase; LVPWs: Thickness of Left Posterior Wall at end-systolic phase; LVAWd: Thickness of Left Ventricular Anterior Wall at end-diastolic phase; LVAWs: Thickness of Left Ventricular Anterior Wall at end-systolic phase; LVDs: Left Ventricular Internal Diameter at end-systolic phase; LVDd: Left Ventricular Internal Diameter at end-diastolic phase. (n=10/group, Two-way ANOVA with Turkey's multiple tests). *: vs 3 m-Saline ($P < 0.05$); †: vs 20 m-Saline ($P < 0.05$).

Supplemental table 11: Primer sequences

Gene (mouse)	Forward Primers (5'-3')	Reverse Primers (5'-3')
Actb	CATTGCTGACAGGATGCAGAAGG	TGCTGGAAGGTGGACAGTGAGG
18s	AGGGGAGAGCGGGTAAGAGA	GGACAGGACTAGGCGGAACA
Gapdh	AGGCCGGTGCTGAGTATGTC	TGCCTGCTTCACCACCTTCT
Fto	GCAGCTGAAATACCCTAAACTG	AGTCTGGTGTTCAAGTACTTGT
Kcne1-mature	CGTTGAAAGGGTCGTGGGAGTG	CGTTGAAAGGGTCGTGGGAGTG
Kcne1-precursor-Left (5'-3')	GGAGGTAGCCAATCATGCTT	
Kcne1-precursor-Internal (5'-3')	TCCTTTCAGGAGTTTTGCTCTGCATC	
Kcne1-precursor-Right (5'-3')	AACAGTCGTGGAATTGGGCA	
**Gene (human)**		
ACTB	GGCACCCAGCACAATGAAG	CCGATCCACACGGAGTACTTG
18s	GGAGTATGGTTGCAAAGCTGA	ATCTGTCAATCCTGTCCGTGT
GAPDH	AGATCCCTCCAAAATCAAGTGG	GGCAGAGATGATGACCCTTTT
FTO	TCAACTGGAAGCACTGTGGAAGAAG	CGAGGCAAGGATGGCAGTCAAG
KCNE1-mature	TGCCCTTCCGCCTTCCACTC	TTTCTCCTCCATCTCAGCCCTGTAC
KCNE1-precursor-Left (5'-3')	GGGTTTTGGCCAATACGTAC	
KCNE1-precursor-Internal (5'-3')	CCTTTAAGAGGTGTGCCTGGGAAGTT	
KCNE1-precursor-Right (5'-3')	TTGGTCAGAAAGGGCGTCAC	

Supplemental table 12: AAV9- shRNA sequences

AAV9	Forward (5'-3')	Reverse (5'-3')
**AAV9-cTnT-Mir30-m-*Fto*-ZsGreen**	GUCAGACCUUCCUAAAGCUTT	AGCUUUAGGAAGGUCUGACTT
**AAV9-cTnT-Mir30-m-*Kcne1*-ZsGreen**	CCGAGAUGACAGCAAGCUATT	UAGCUUGCUGUCAUCUCGGTT
**AAV9-cTnT-Mir30-m-nc-ZsGreen**	UUCUCCGAACGUGUCACGUTT	ACGUGACACGUUCGGAGAATT
